# (Ro)vibrational Spectroscopic Constants, Lifetime and QTAIM Evaluation of Fullerene Dimers Stability

**DOI:** 10.3390/molecules28135023

**Published:** 2023-06-27

**Authors:** Rodrigo A. Lemos Silva, Mateus R. Barbosa, Caio R. Martins, Daniel F. Scalabrini Machado, Luciano Ribeiro, Heibbe C. B. de Oliveira, Demétrio A. da Silva Filho

**Affiliations:** 1 Instituto Federal de Educação, Ciência e Tecnologia de Goiás (IFG), Câmpus Jataí, Jataí 75804-714, GO, Brazil; rodrigo.aparecido@ifg.edu.br; 2 Grupo de Química Teórica e Estrutural de Anápolis (GQTEA), Campus Central Anápolis de Ciências Exatas e Tecnológicas Henrique Santillo, Universidade Estadual de Goiás, CP 459, Anápolis 75132-903, GO, Brazil; caiorodriguesmartins@hotmail.com; 3 Laboratório de Estrutura Eletrônica e Dinâmica Molecular (LEEDMOL), Instituto de Química, Universidade Federal de Goiás, CP 131, Goiânia 74001-970, GO, Brazil; mateusrodriguesbarbosa@gmail.com; 4 Laboratório de Modelagem Molecular de Sistemas Complexos (LMSC), Instituto de Química, Universidade de Brasília, CP 4478, Brasília 70919-970, DF, Brazil; 5 Grupo de Semicondutores Orgânicos, Instituto de Física, Campus Darcy Ribeiro, Universidade de Brasília, CP 4478, Brasília 70919-970, DF, Brazil; dasf@unb.br

**Keywords:** fullerene dimers, spectroscopic properties, Dunham, DVR, QTAIM, lifetime

## Abstract

The iconic caged shape of fullerenes gives rise to a series of unique chemical and physical properties; hence a deeper understanding of the attractive and repulsive forces between two buckyballs can bring detrimental information about the structural stability of such complexes, providing significant data applicable for several studies. The potential energy curves for the interaction of multiple van der Waals buckyball complexes with increasing mass were theoretically obtained within the DFT framework at ωB97xD/6−31G(d) compound model. These potential energy curves were employed to estimate the spectroscopic constants and the lifetime of the fullerene complexes with the Discrete Variable Representation and with the Dunham approaches. It was revealed that both methods are compatible in determining the rovibrational structure of the dimers and that they are genuinely stable, i.e., long-lived complexes. To further inquire into the nature of such interaction, Bader’s QTAIM approach was applied. QTAIM descriptors indicate that the interactions of these closed-shell systems are dominated by weak van der Waals forces. This non-covalent interaction character was confirmed by the RDG analysis scheme. Indirectly, QTAIM also allowed us to confirm the stability of the non-covalent bonded fullerene dimers. Our lifetime calculations have shown that the studied dimers are stable for more than 1 ps, which increases accordingly with the number of carbon atoms.

## 1. Introduction

Fullerenes, also known as buckyballs, are hollow polyhedral molecules constituted of sp2-hybridized covalently bonded carbon atoms, with the faces made up of a combination between pentagons and hexagons [1,2,3]. They were originally predicted by Osawa in 1970 [1] and first synthesized in 1985 by Kroto and co-workers [2]. The iconic caged shape of buckyballs gives rise to interesting chemical properties, such as trapping small molecules inside fullerenes [4,5], organometallic fullerenes acting as hydrogen adsorbents [6], and endohedral doping with metals resulting in high electrical conductivity [7]. The unique physical and chemical properties of fullerenes attracted a lot of attention aimed at their possible applications to organic electronics. For example, as components of photovoltaic cells [8,9], for the development of sensors employed for the capture of pollutants [10,11], or for electron transport through self-assembled monolayers of functionalized fullerenes [12,13]. In this context, information about the intermolecular potential between fullerene molecules, the equilibrium distance, and energy minima of the van der Waals contacts are of great importance to understanding these phenomena [14].

Dynamic force microscopy associated with dispersion-corrected density functional theory (DFT) was used to map the intermolecular potential of the *C*_60_ fullerene by Sweetman and co-workers, showing that the different orientations of the *C*_60_ introduced a variation from ~50 to 60 meV in the potential minimum [15]. These authors also showed that there is a subtle interplay between attractive and repulsive forces governing the intermolecular potential, in which variations of the dispersion interaction are screened by repulsive interaction even at large distances.

In one of our previous manuscripts, we investigated the influence that the different orientations of dimers of *C*_70_ have on the potential energy curve and in the spectroscopic constants [16]. It was shown that different configurations might be accessed by a fullerene dimer, which may change the spectroscopic and stability characteristics of those molecules. Furthermore, in another of our recent research [11], the importance of non-covalent interactions (NCI) was investigated to better understand the characteristics of the intermolecular interaction between the C_59_X doped fullerene (X = B, Al, Ga, Si, Ge, N, and P) and the emerging pollutant Carbamazepine CBZ. In this research, it was shown that intermediate stable states could also be accessed due to a small change in the configuration of the interaction, allowing the discovery of new active sites for CBZ adsorption on C_59_X fullerenes.

Therefore, a deeper understanding of these features concerning NCI, as well as the spectroscopic properties and structural stability of the van der Waals bonds provided by theoretical data, are of great significance when studying supramolecular fullerenes for several applications. Baggio and co-workers [17] have reported a theoretical study of the binding energies between ammonia and metallo-phthalocyanines, in which the rovibrational spectroscopic constants were obtained by approximating both species as a huge “monoatomic” compound. Such an approach neglects all intramolecular interactions, thus greatly simplifying the solution of the nuclear Schrödinger equation while reporting good correlation with experimental data, characterizing the spontaneity of ammonia/metallo-phthalocyanines complexes at temperatures around 500 K. The same protocol was applied, by the same authors, to predict the thermodynamics and stability of methanol noble gasses complexes [18].

**Figure 1:**
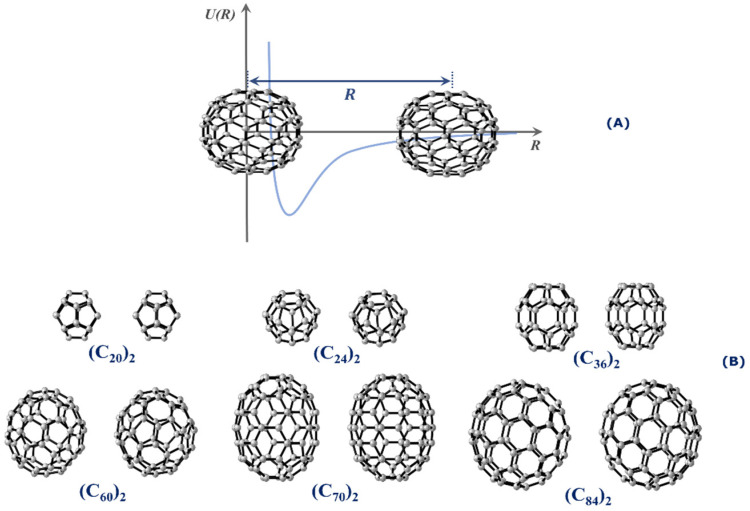
(**A**) Schematic illustration of the rigid scan used to construct the PEC of the interaction of the fullerene dimers whose interaction coordinate is along the center-to-center distance separated by *R*. (**B**) Relative orientations between the fullerene dimers considered in the PEC calculations.

This work aims at calculating the potential energy curves (PECs) of numerous fullerene dimers of different sizes. Specifically, we are interested in studying the stability and determining the spectroscopic properties of (*C*_20_)_2_, (*C*_24_)_2_, (*C*_36_)_2_, (*C*_60_)_2_, (*C*_70_)_2_, and (*C*_84_)_2_. All dimers employed in this research are presented in Figure 1. Spectroscopic properties constitute an interesting tool to assess the stability of the dimer form because long-lived complexes tend to oscillate around the equilibrium distance, and therefore, quantized (ro)vibrational levels are expected to exist in the potential well. To achieve this goal, we employed the same methodology applied in the calculation of spectroscopic properties of diatomic systems [16,19,20,21] to the case of fullerene dimers, as will be further discussed in this article. The stability of these fullerene dimers was also investigated through lifetime calculations, while the quantum theory of atoms in molecules (QTAIM) [22] was employed to determine the dimers’ type of intermolecular interaction. 

## 2. Results and Discussion

**Figure 2:**
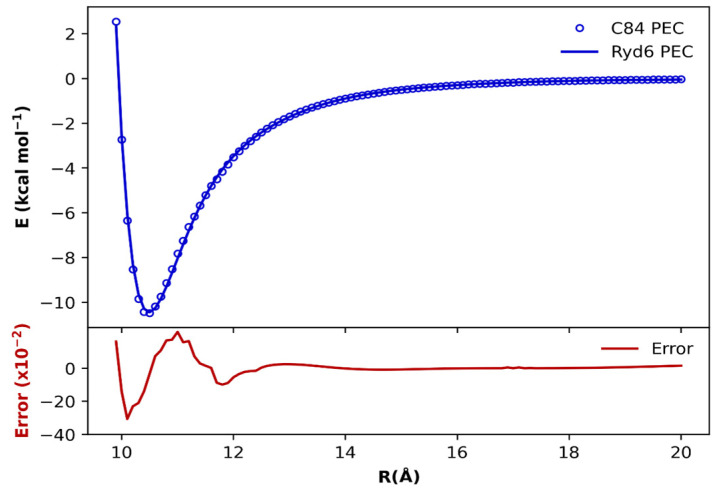
Potential energy curves (**top**) fitted with the analytic Rydberg function for the (*C*_84_)_2_ dimer calculated at the ωB97xD/6−31G(d) level of theory. The point-by-point error ((**bottom**) in kcal mol^−1^) is also plotted to magnify the overall fit accuracy in the entire range of internuclear separation.

Let us begin our discussion by presenting the PECs obtained for the (*C*_84_)_2_ fullerene dimer in Figure 2 and for the remaining dimers in Figures S1–S6. As discussed in the Methodology section, the ab initio ωB97xD/6−31G(d) points (blue dots in Figure 2) were fitted through the Ryd6 analytical function (blue line in Figure 2). The quantitative point-by-point difference between ab initio energies and fitted Ryd6 PEC accounts for the error in the fitting procedure (red line in Figure 2). The optimized Ryd6 parameters for each fullerene dimer are reserved in Table S1 and Table S2 to avoid the proliferation of the results. Notice from Figure 2 that the fitting errors do not surpass 0.05 kcal mol^−1^ for the (*C*_84_)_2_ dimer. The same fitting accuracy was transferable among the other fullerene dimers, indicating that the optimization of the Ryd6 parameters is consistent and reliable, as confirmed by the global accuracy of the χ^2^ values in the range of 10^−5^ kcal mol^−1^ to 10^−4^ kcal mol^−1^ (see Table S1 and Table S2). 

**Figure 3:**
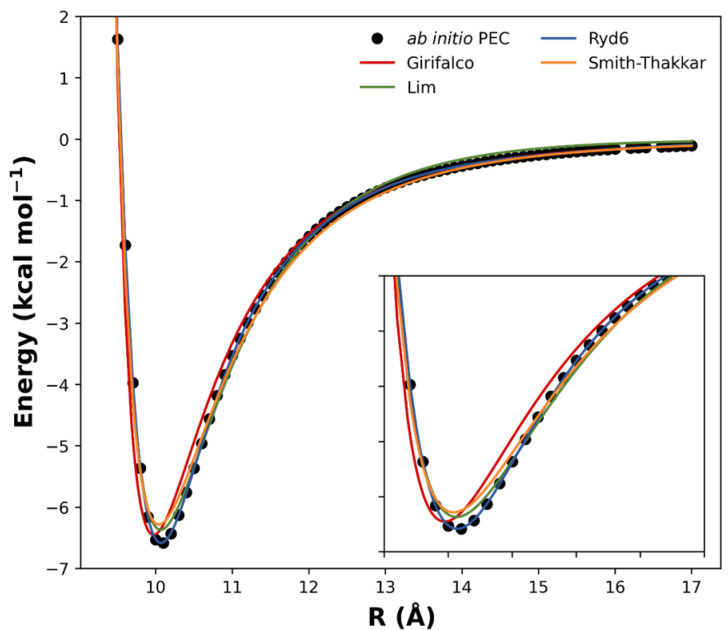
Comparison between the fitted analytical potentials for the (*C*_60_)_2_ dimer.

Other published analytical potentials were also evaluated for the (*C*_60_)_2_ and compared to the Ryd6 potential. The intermolecular potential forms of Girifalco, Smith–Thakkar, and Lim were fitted to the ωB97xD/6−31G(d) points (Figure 3), and their corresponding expressions were reserved in Table S5 along with their optimized parameters and squared correlation effects. Originally, these analytic forms were proposed to fit bulk data obtained from crystals, neglecting deformation and orientation effects [23]. Although deformation can be neglected in such types of van der Waals complexes, orientation effects should play an important role in the intermolecular interaction of carbon nanostructures [16]. Furthermore, we see in Figure 3 that the potential forms of Girifalco, Smith–Thakkar, and Lim were unable to describe the PEC at the equilibrium region. Since the NSE depends on a good description of the PEC, these potential forms were not effectively suitable to describe the spectroscopic constants for the fullerene dimers studied herein. 

**Figure 4:**
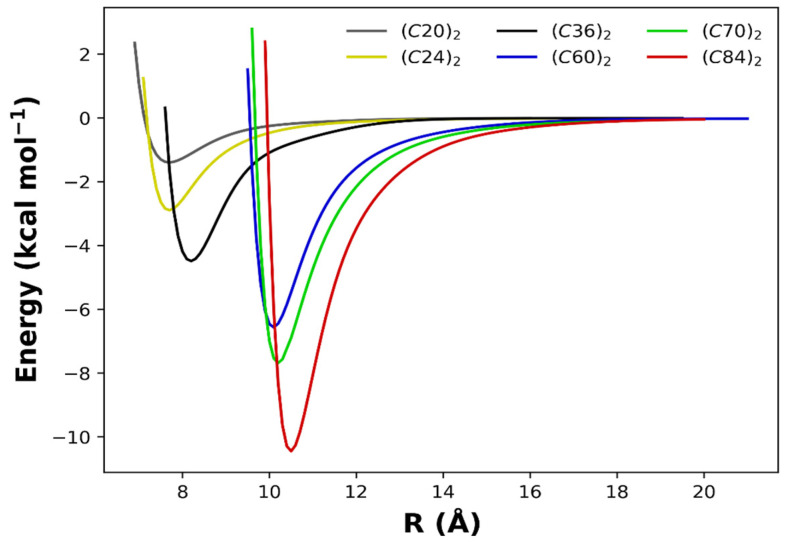
Potential energy curves modeled by the analytic Rydberg function for the fullerene dimers calculated at the ωB97XD/6−31G(d) chemical model.

To better appreciate the overall aspects of the obtained PECs, we present in Figure 4 the superimposed intermolecular potential for all dimers studied in this work. Here, we are eager to explore the size effects on the potential energy minimum (*D_e_*) and its separation (*R_e_*) explicated in Table 1, along with other results obtained with different methods for the (*C*_60_)_2_ and the (*C*_70_)_2_ dimers. From Figure 4 and Table 1, it is noticed that as the number of carbon atoms in each monomer increases, the intermolecular interaction is intensified, and the potential well becomes more profound.

We now focus on the results obtained by solving the NSE (Equation (4)). The values of the spectroscopic constants obtained using the Dunham and DVR methods are presented in Table 2. There was a satisfactory agreement between the values of the spectroscopic constants through both methods. The rotational constant *B_e_* and the rotor-vibrating coupling constant *α_e_* calculated for the dimers were negligibly small due to the relatively large reduced mass for the studied systems. The reduced mass *μ* values were taken to be 120.11u, 144.13 *u*, 216.19 *u*, 360.33 *u*, 420.38 *u,* and 504.46 *u* for the (*C*_20_)_2_, (*C*_24_)_2_, (*C*_36_)_2_, (*C*_60_)_2_, (*C*_70_)_2_, and (*C*_84_)_2_ dimer, respectively. Concerning the fundamental vibrational frequency *ω_e_*, we can see from Table 2 that there was a small blue shift (less than ~10 cm^−1^) when increasing the size of the dimers. By analyzing the anharmonicity effects measured by the *ω_e_x_e_* constant, we notice that it becomes more relevant for the smallest dimers, representing one-tenth of the harmonic frequency for the (*C*_20_)_2_ and (*C*_24_)_2_ dimers.

The vibrational eigenvalues (with *J* = 0) of the NSE obtained through the DVR method are presented in Table 3 for the lowest-lying vibrational states of the fullerene dimers. Because of the small rotational effects on the interaction mode (*B_e_* ~10^−4^ cm^−1^ and *α_e_* ~10^−7^ cm^−1^), the dimers are essentially a rigid rotator system; therefore, the values for *ε_υ_*_,*J*_ with *J* ≠ 0 were suppressed in Table 3. Additionally, we estimated the maximum number of vibrational levels harbored by the PECs for each fullerene dimer, i.e., we determined the largest value of υ in *ε_υ_*_,*J*_ satisfying the inequality *ε_υ_*_,*J*_ < *D_e_*. It turns out that the number of bound vibrational states for each dimer was 60, 70, 146, 222, 237, and 265 for the ${\left(C_{20}\right)}_{2},$ ${\left(C_{24}\right)}_{2}$, ${\left(C_{36}\right)}_{2}$, ${\left(C_{60}\right)}_{2}$, ${\left(C_{70}\right)}_{2}$, and ${\left(C_{84}\right)}_{2}$ systems, respectively.

**Figure 5:**
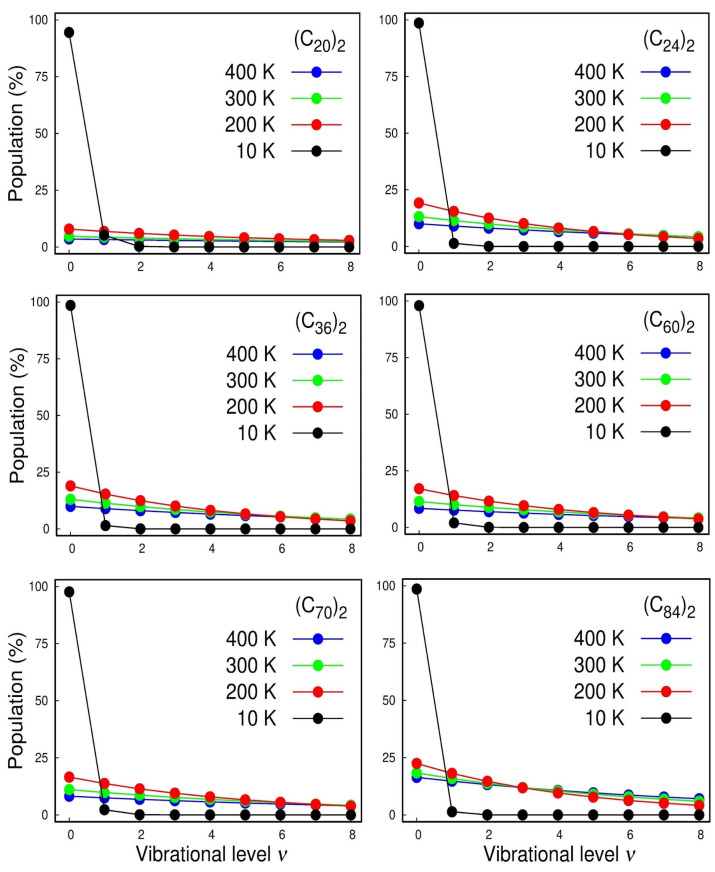
Population analysis of the lowest-lying vibrational levels for the ${\left(C_{20}\right)}_{2},$, ${\left(C_{24}\right)}_{2}$, ${\left(C_{36}\right)}_{2}$, ${\left(C_{60}\right)}_{2}$, ${\left(C_{70}\right)}_{2}$, and ${\left(C_{84}\right)}_{2}$ dimers.

With the rovibrational constants in hand, we can also calculate the vibrational population for each state and the temperature effects of the Boltzmann distribution of the quantized levels, which are displayed in Figure 5. The calculated spectroscopic constants were inserted in the rovibrational partition function that can be found in ref. [17]. 

In Figure 5, we first focused on the cases of the strongest interacting systems, i.e., ${\left(C_{70}\right)}_{2}$ and ${\left(C_{84}\right)}_{2}$ where we can see the anharmonicity of the vibrational structure. For instance, at room temperature (300 K), we see that, even for those strongest interacting systems, less than ¼ of the dimers lie on the vibrational ground state and that the inclusion of higher vibrational excited states is mandatory for a good description of the normal modes. The harmonic oscillator accurately models these systems only at very low temperatures of ~10 K. Therefore, once again, it highlighted the importance of using adjustable potential functions, such as Ryd6, to adjust the potential energy curve employed in NSE to achieve a suitable description of the system’s spectroscopic constants.

**Figure 6:**
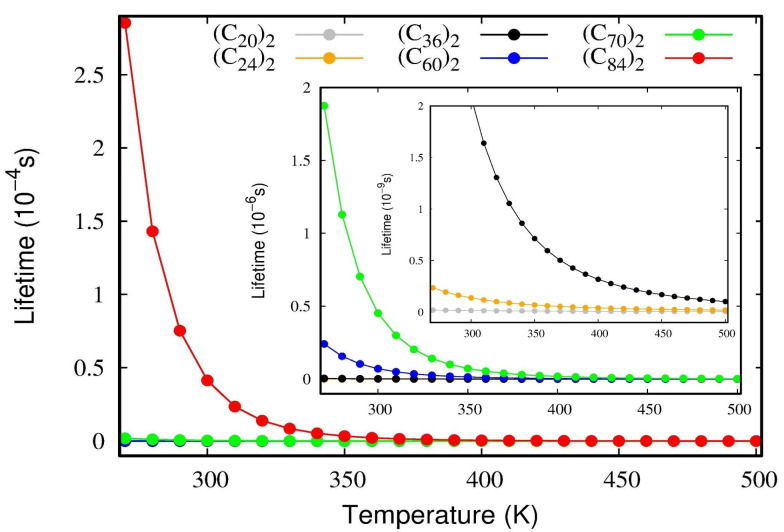
The lifetime of fullerene dimers as a function of temperature obtained using Equation (10). The inset plot magnifies the lifetimes for the (*C*_20_)_2_ and (*C*_24_)_2_ dimers.

To confirm the stability of these dimers, a lifetime calculation was performed using the *D_e_* and *ω_e_* parameters occurring in Equation (11). The lifetimes of the van der Waals complexes as a function of temperature are presented in Figure 6. As a general rule, when a given complex presents lifetimes larger than 1.0 ps, it is deemed to be a stable one; on the other hand, if the value is inferior to this threshold value, the complex is considered transitory [29]. Figure 6 reveals that all fullerene dimers studied here present lifetimes that are larger than 1.0 ps, even when the temperature is raised to normal conditions. The numerical values are reserved in Table S4 for temperatures ranging from 200 to 500 K. The inset plot in Figure 6 shows that even the weakest complexes (*C*_20_)_2_ and (*C*_24_)_2_ are of the order of *τ*~1 ps, thus relatively stable. Figure 6 also confirms that the lifetime values have the same trends observed for the interaction energies, *D_e_*, and therefore (*C*_84_)_2_ is the most stable system, followed by (*C*_70_)_2_, (*C*_60_)_2_, (*C*_36_)_2_, (*C*_24_)_2,_ and (*C*_20_)_2_.

Understanding the nature of the NCIs occurring in van der Waals complexes is of primary interest to assess the stability of the dimers and to gain insight into the physical nature of the interaction. To guarantee accurate NCI description, the fullerene dimers were optimized at the equilibrium distance obtained from the PECs calculations. Although the rigid scan showed acceptable energies, a dimer structure optimized at the minimum energy should be considered, especially for the analysis of the interaction type between the monomers. The QTAIM approach developed by Bader [22] is a qualitative quantum chemical tool to evaluate the NCIs from an analysis of the topological properties of the electron density. Within the QTAIM methodology, the so-called bond paths describe a line of local maximum electron density connecting two nuclear critical points (NCPs, i.e., the actual position of the atoms) that interacts either covalently or non-covalently. For this purpose, the electron density (ρ) and its Hessian matrix ${(\nabla }^{2}\rho =\lambda_{1}+\lambda_{2}+\lambda_{3})$ were analyzed at the region between two interacting molecules. The algebraic signs of the eigenvalues $\lambda_{i}$ (i=1,2,3) of the Hessian matrix distinguish the nature of the critical points. At the nuclei, all eigenvalues are negatives. At this region, the density exhibits local maximum, the so-called nuclear critical point (NCP, i.e., the atoms’ actual position). When two eigenvalues of the Hessian matrix are negative ($\lambda_{1},\lambda_{2})$, we observed the formation of a bond critical point (BCP). When only one value of the Hessian matrix is negative ($\lambda_{1})$, it is observed as a ring-critical point (RCP). Generally, RCP are points of steric effects. When none of the eigenvalues are negative, the formation of a local minimum in the density, named cage critical point (CCP), is observed. At the BCP, the values of ρ and $\nabla^{2}\rho$ are closely related to the nature of the interaction.

**Figure 7:**
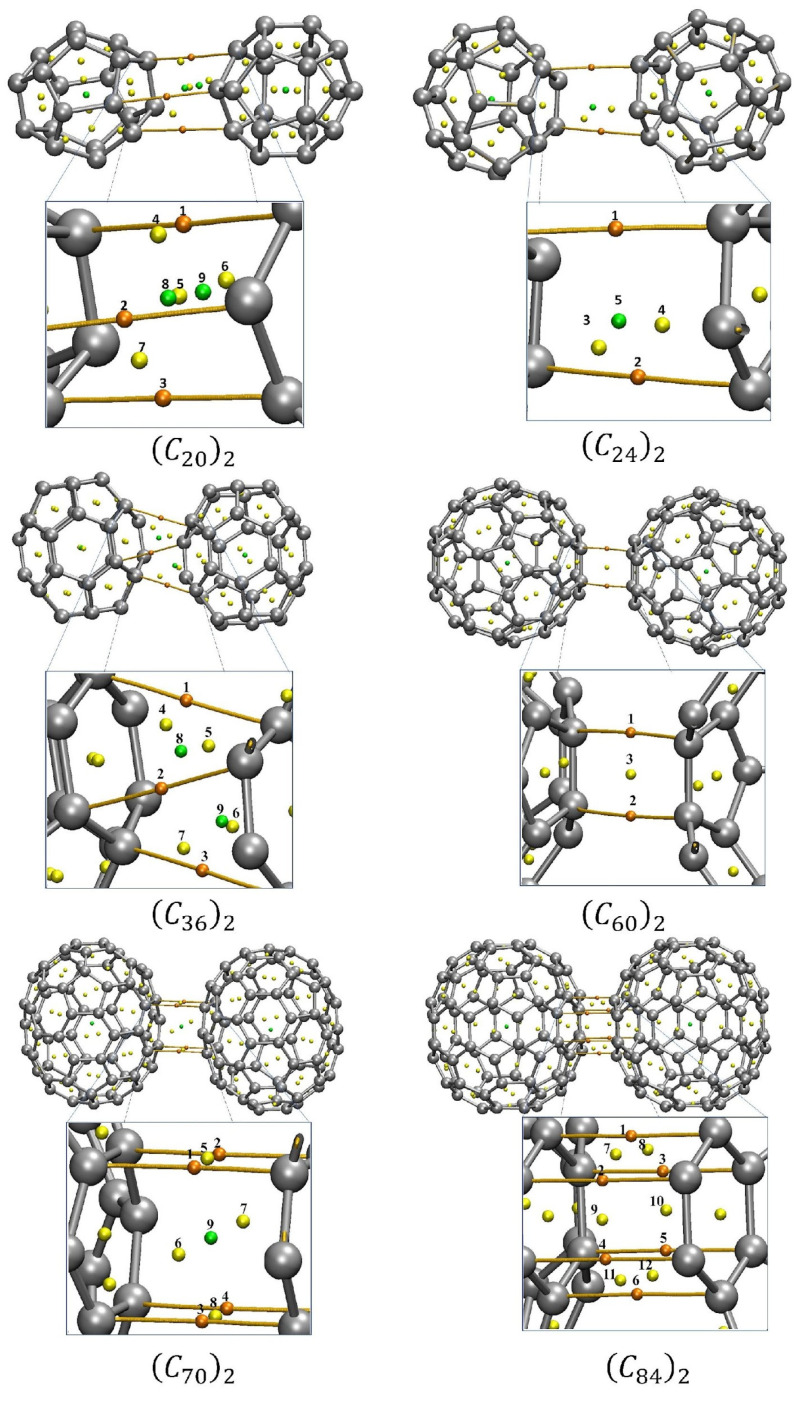
QTAIM critical points for the fullerene dimers. The orange dots represent the BCPs between the molecules, the yellow dots represent the critical points of the RCP ring, and the green dots represent the cage critical points of CCP. Table S6 presents the values of the Electron density *ρ*_cp_ (in *e/a_0_^3^*), the Laplacian of the electron density ∇^2^*ρ*_cp_ (in *e/a_0_*^5^) and the total energy H_BCP_ (in Hartree). To facilitate the view and comparation with the results shown the Table S6, the CP where numbered. The QTAIM analysis was performed for the optimized dimers at the equilibrium position.

Figure 7 portrays the molecular graphs of the interacting fullerene dimers separated at the minimum of the well showing the BCPs, RCPs, CCPs, and bond paths. Interestingly, for those fullerenes that were more spherically symmetric, we observed fewer CPs and bond paths connecting the two (*C*_n_)_2_ monomers. 

To facilitate the analyses and interpretation of the NCIs, QTAIM offers a relatively simple and straightforward interpretation of the intermolecular forces by calculating parameters at the BCP, such as the electron density (*ρ*_BCP_), its Laplacian (∇^2^*ρ*_BCP_) and the total electron energy density (H_BCP_), which are summarized in Table S6. The nature of the interacting BCP can be categorized depending on the signal and magnitude of ∇^2^*ρ*_BCP_ and H_BCP_. When ∇^2^*ρ*_BCP_ < 0 and H_BCP_ < 0, it is a good indicator of a very strong interaction typical of open-shell interactions (covalent bonds). On the other hand, when ∇^2^*ρ*_BCP_ > 0 and H_BCP_ > 0, the BCP along the bond path is commonly associated with closed-shell interactions (NCIs) when accompanied by a small value of *ρ*_BCP_ [30,31,32].

From Table S6, we observe small values of *ρ*_BCP_ at the BCPs between the monomers of the order of $10^{-3}e\;{a_{0}}^{-3}$, and associated with the positive values of ∇^2^*ρ*_BCP_ and H_BCP._ This confirms, from a QTAIM standpoint, that the intermolecular interactions can be weak closed-shell interactions, i.e., they attract each other through van der Waals forces.

**Figure 8:**
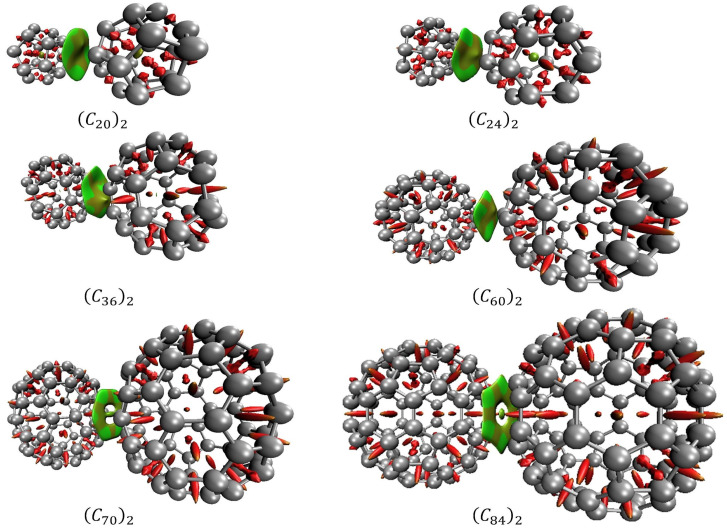
Non-covalent interactions for the fullerene dimers obtained by RDG. Green-colored and light-brown isosurfaces show non-covalent interactions, while red-colored isosurfaces present steric effects.

In association with QTAIM, the analysis of the reduced density gradient (RDG) aids the interpretation of the nature of the NCIs. RDG introduces a generalization of the approach focused on critical points. This allows the production of isosurfaces that yield additional evidence for non-covalent interactions. Figure 8 presents the diagrams of isosurfaces for NCIs present in the fullerene dimers. In Figure 8, green-colored isosurfaces show non-covalent interactions, while red-colored isosurfaces present steric effects. 

In the RDG analysis, while the behavior of s indicates the critical points in a 2D graph, the combination of electron density ρ and the signal of $\lambda_{2}$ provides a tool to distinguish between repulsive or attractive interactions. For $\lambda_{2}<0$ it is reported as an attractive interaction, while for repulsive interaction, $\lambda_{2}>0$ [32]. For this reason, a real space function, ${sign(\lambda }_{2})\rho$, namely the product of $\lambda_{2}$ with ρ is employed to help with this task of identifying the type of the interaction. In summary, large negative values of ${sign(\lambda }_{2})\rho$ are indicative of strong attractive interactions, such as hydrogen bounds or dipole–dipole interactions. Conversely, large positive values of ${sign(\lambda }_{2})\rho$ represents non-bonding interactions, and when ${sign(\lambda }_{2})\rho$ ≈ 0, it is indicative of van der Waals interactions [30,32]. 

**Figure 9:**
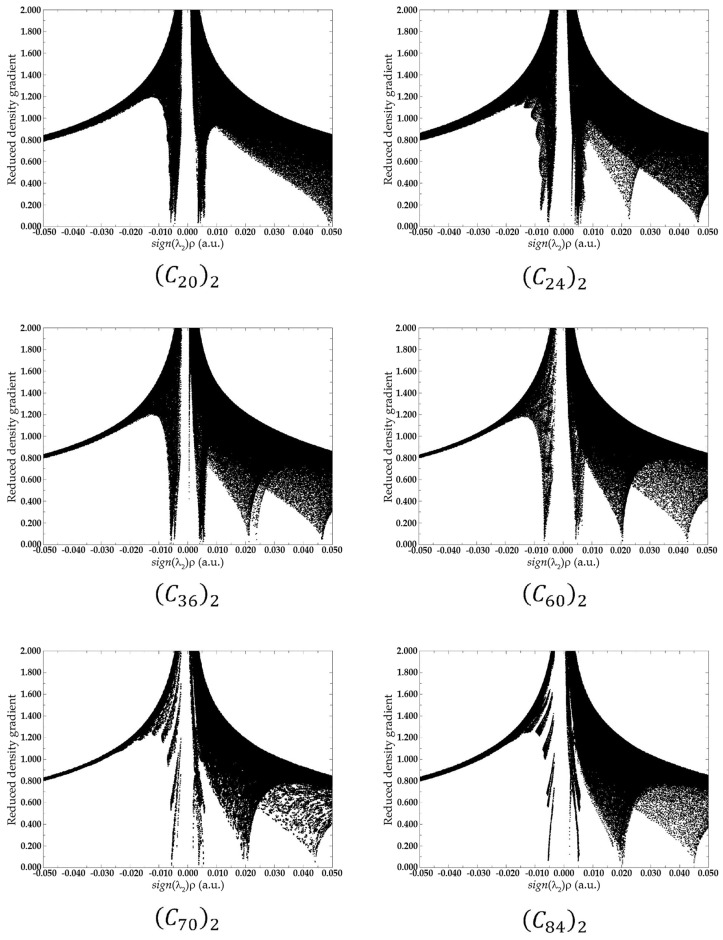
Scatter graph with the plots of ${sign(\lambda }_{2})\rho$ versus RDG of non-covalent interaction for fullerenes dimers. The two picks shown in the medium of the X-axis in the graph are indicative of the van der Waals interaction between the two fullerenes. All data were obtained by evaluating ωB97XD/6-31G(d) density.

Figure 9 presents the scatter graph of ${sign(\lambda }_{2})\rho$ versus RDG of non-covalent interaction for fullerenes dimers. In the graph, the regions where the values of the function ${sign(\lambda }_{2})\rho$ are between −0.010 and 0.010, reveal van der Walls interactions. Those regions are the spikes located on the X-axis in the graph and are represented by the green and light brown isosurfaces shown in Figure 8. Regions where ${sign(\lambda }_{2})\rho$ assumes values over 0.020 are regions of steric effects. Those regions are represented by the red surfaces in Figure 8. 

QTAIM results and RDG index showed electron density depletion in the intermolecular region. The analyses of the electronic density, the Laplacian of the electronic density, and the total energy at the critical points in association with the RDG isosurfaces and with the spikes observed in the scattering graphs, presented in Figure 8 and Figure 9 confirm the non-covalent interaction as the responsible for the fullerene dimer stabilization. This van der Waals nature of the NCIs correlates very well with the values of the potential depth obtained by our DFT estimates. 

In addition, QTAIM can be employed as an indirect tool to evaluate the stability of weak interacting molecules [33,34,35,36]. When an unstable interaction is analyzed under the QTAIM schemes, the presence of critical degenerate points can be observed in the intermolecular region. The closer the RCP and the BCP are, the more unstable the interaction tends to be. In unstable intermolecular interactions, a small energy disturbance can cause the breaking of an RCP to form a BCP [22]. As can be seen in the QTAIM parameters, shown in Table S6, no degenerated critical point was observed. This way, the QTAIM analyses deal with energetic and lifetime evaluation, confirming the fullerene dimers’ stability, in particular for the (*C*_70_)_2_ and (*C*_84_)_2_ systems which showed a well-defined face-to-face interaction, presented for stable and direct bond paths in the intermolecular region. 

Finally, the NCI analyses provided by QTAIM and RDG approach allow us to verify the interacting more favorable face for each fullerene dimer as well the trend of the bond. It was observed by QTAIM analysis that the dimers of C_36_, C_70,_ and C_84_ fullerenes are prone to interact through their hexagonal–hexagonal faces. The RDG isosurfaces confirm this trend through its hexahedron form. The (*C*_60_)_2_ dimers presented an interaction via one pentagonal face oriented to an inter-pentagon bonding (see Table S8 in supporting information and Figure S7). Although this configuration differs from the more energetically stable configuration observed by Sharapa and co-authors’ work [23], it is notable that our calculations returned that these pentagonal faces are oriented towards the configuration of inter-pentagon connections. Similar inter-pentagon relative orientation has been observed for bulk C_60_ fullerenes at low temperatures [18].

## 3. Methods

Intermolecular Interaction Potential. To determine the PECs for all systems, we performed a rigid scan on the interaction coordinate, i.e., keeping one of the monomers stationary and then moving away the second monomer with incremental steps of 0.1 Å with respect to the center-to-center distance, as shown in Figure 1A. The binding energies ($E_{Binding}$) were estimated by means of the supermolecular approach given by [37],

(1) $$E_{Binding}=E_{dimer}-\left(E_{mon,A}+E_{mon,B}\right),$$

where $E_{dimer}$ is the energy of the fullerene dimer, $E_{mon,A}$ and $E_{mon,B}$ represent the energy of monomers A and B, respectively. Figure 1B shows the relative orientations of each fullerene dimer used to build the targeted PECs. The structures for each fullerene shown in Figure 1B were retrieved from the $C_{n}$ fullerenes database [38]. We selected the $C_{20}$ (I_h_), $C_{24}$ (D_6d_), $C_{36}$ (D_6h_), $C_{60}$ (I_h_), $C_{70}$ (D_5h_), and $C_{84}$ (D_2_) fullerenes because they presented the lowest energy in the ground state and the lowest strain energy being considered as the more stable isomers [39,40].

Electronic structure calculations were performed using DFT at the ωB97xD/6−31G(d) chemical model [23,24], as implemented in the Gaussian 09 package [41]. The range-separated hybrid functional ωB97xD was chosen because it is dispersion-corrected through Grimme’s GD2 empirical dispersion model [42] and has shown to be very reliable in capturing the binding energy of the C_60_ dimer when Grimme’s empirical dispersion model GD3 is used [23]. Grimme’s GD2 model has an extra energy term that includes the pair-wise London dispersion interaction between the monomers and uses a similar damping function to that used by the GD3 model. Basis Set Superposition Error (BSSE) correction was also included through the counterpoise method of Boys and Bernardi [43].

PECs were then modeled by fitting the ab initio points using the 6th degree Rydberg analytical function (termed here as Ryd6) [44], which is written as,

(2) $$U\left(R\right)=-D_{e}\left(1+\sum_{j=1}^{6}a_{j}{\left(R-R_{e}\right)}^{j}\right)e^{-a_{1}\left(R-R_{e}\right)},$$

where $a_{j}$ are adjustable coefficients, and *R_e_* represents the equilibrium position determined from quantum-chemical calculations. The parameters were optimized employing the Generalized Simulated Annealing (GSA) [45,46] method, required to minimize the cost function given by

(3) $$\chi^{2}=\frac{1}{N_{p}}\sum_{i=1}^{N_{p}}{\left(E_{ab,i}-E_{theo,i}\right)}^{2},$$

where $E_{ab,i}$ corresponds to the ab initio energies, $E_{theo,i}$ is the fitted energy and $N_{p}$ is the number of points. 

Rovibrational Spectroscopic Constants. After the complete description of the PECs of the interacting dimers, we solved the nuclear Schrödinger equation (NSE) to obtain its dynamic properties. For the fullerene dimers studied here, the radial NSE, under the Born–Oppenheimer approximation, is given by

(4) $$\frac{-1}{2\mu }\frac{\partial^{2}F\left(R\right)}{\partial R}+U_{ef}\left(R\right)F\left(R\right)=\varepsilon_{\upsilon ,J}F\left(R\right),$$

where $F\left(R\right)=R\Psi_{\upsilon ,J}\left(R\right)$ and μ is the reduced mass of the system. The term $U_{ef}\left(R\right)$ in Equation (4) is the effective potential:

(5) $$U_{ef}\left(R\right)=\frac{J\left(J+1\right)}{2\mu {R_{e}}^{2}}+U\left(R\right),$$

which includes the intermolecular potential $U\left(R\right)$ and centrifugal distortions due to the coupling with the rotational levels. By solving the NSE given by Equation (4), we are considering the dimers as two “large atoms”, i.e., we are neglecting all intramolecular effects in an analogy to the methodology of Baggio and co-workers [17].

The spectroscopic constants of the fullerene dimers were obtained employing two different approaches. The first approach consists in solving the NSE to obtain its eigenvalues and then inserting them in a set of equations for the spectroscopic constants as described in refs [20,21,47].

(6) $$\begin{array}{c} \omega_{e}=\frac{1}{24}\left[141\left(\varepsilon_{1,0}-\varepsilon_{0,0}\right)-93\left(\varepsilon_{2,0}-\varepsilon_{0,0}\right)+23\left(\varepsilon_{3,0}-\varepsilon_{1,0}\right)\right];\\ \omega_{e}x_{e}=\frac{1}{4}\left[13\left(\varepsilon_{1,0}-\varepsilon_{0,0}\right)-11\left(\varepsilon_{2,0}-\varepsilon_{0,0}\right)+3\left(\varepsilon_{3,0}-\varepsilon_{0,0}\right)\right];\\ \omega_{e}y_{e}=\frac{1}{6}\left[3\left(\varepsilon_{1,0}-\varepsilon_{0,0}\right)-3\left(\varepsilon_{2,0}-\varepsilon_{0,0}\right)+3\left(\varepsilon_{3,0}-\varepsilon_{2,0}\right)\right];\\ \alpha_{e}=\frac{1}{8}\left[-12\left(\varepsilon_{1,1}-\varepsilon_{0,1}\right)+4\left(\varepsilon_{2,1}-\varepsilon_{0,1}\right)+4\omega_{e}-23\omega_{e}y_{e}\right];\\ \gamma_{e}=\frac{1}{4}\left[-2\left(\varepsilon_{1,1}-\varepsilon_{0,1}\right)+\left(\varepsilon_{2,1}-\varepsilon_{0,1}\right)+2\omega_{e}x_{e}-9\omega_{e}y_{e}\right]. \end{array}$$

In this work, the NSE was solved using the Discrete Variable Representation (DVR) method [47]. The spectroscopic constants given in Equation (6) are the harmonic frequency $\omega_{e}$, the anharmonicity constants $\omega_{e}x_{e}$ and $\omega_{e}y_{e}$ $\alpha_{e}$ and $\gamma_{e}$ are the rovibrational coupling constants. 

The second approach is the method developed by Dunham [48]. In Dunham’s method, the spectroscopic constants are calculated by taking derivatives of higher orders of the PEC expanded as a Taylor series around the equilibrium distance *R_e_*. The second (*d*_2_), third (*d*_3_), and fourth (*d*_4_) order derivatives of U(*R*) equate to the following spectroscopic constants [15].

(7) $$d_{2}=4\pi^{2}\mu c^{2}\omega_{e}^{2},$$

(8) $$d_{3}=\frac{-3d_{2}}{R_{0}}\left(1+\frac{\alpha_{e}\omega_{e}}{6B_{e}^{2}}\right),$$

and

(9) $$d_{4}=\frac{d_{2}}{R_{0}^{2}}\left[15\left(1+\frac{\alpha_{e}\omega_{e}}{6B_{e}^{2}}\right)-\frac{8\omega_{e}x_{e}}{B_{e}}\right].$$

Rovibrational population analyses as a function of the temperature were conducted using Boltzmann statistics, implementing a rovibrational partition function to account for the non-rigid rotator and anharmonic effects. More details about the explicit form of the partition function are provided in ref. [17]. The rovibrational level occupations were assessed in the range of 10–400 K.

Lifetime of the van der Waals complexes. Slater’s theory [49] was employed to measure the lifetime of the dissociative process of the fullerene dimers. The unimolecular dissociation of the complex is supposed to occur when the interaction coordinate reaches the dissociation energy threshold, *D_e_* [50]. If the complex has enough energy to reach *D_e_*, the frequency of dissociation becomes simply the vibrational frequency, *ω_e_*, of the interacting molecules, and the dissociation rate constant becomes:

(10) $$k\left(t\right)=\omega_{e}e^{\frac{-D_{e}-\varepsilon_{0,0}}{RT}},$$

where *R = N_A_k_b_* is the ideal gas constant, and *ε*_0,0_ is the rovibrational ground state of the fullerene dimer. The reciprocal of Equation (10) renders the dissociation lifetime of the complexes.

(11) $$\tau \left(t\right)=\frac{1}{\omega_{e}}e^{\frac{D_{e}-\varepsilon_{0,0}}{RT}}.$$

According to Slater’s theory, lifetime estimations are only valid for systems under high/intermediate pressures.

QTAIM Characterization of the NCIs. To investigate the type of intermolecular interaction, we take advantage of the topological analysis provided by Bader’s theory, QTAIM [51,52,53]. In the QTAIM approach, every topological idiosyncrasy of *ρ*(*r*) (whether a maximum, minimum, or saddle point) is associated with a specific point in space, referred to as the critical point, where the gradient of *ρ*(*r*) vanishes [51]. Currently, the QTAIM approach has found large applicability in the study of many electronic properties; specifically, it has been employed in the study of weak interacting systems [10,11,54]. The topological characterization of the NCIs was studied in the case of fullerene dimers. The NCIs were classified according to the topological properties at the Bond Critical Point (BCP): the electron density (*ρ*(*r*)), Laplacian of electron density (∇^2^*ρ*(*r*)), and energy density (H_CP_)). All QTAIM properties were computed using the wavefunction analysis program Multiwfn.

Reduced density gradient characterization of the NCIs. In association with QTAIM, reduced density gradient (RDG) [30,32] was employed to characterize the NCIs. The RDG analysis provides a dimensionless index, presented in Equation (12), called reduced density gradient, s, which is based in the electron density *ρ*(*r*) and its derivate (∇*ρ*(*r*)) [32].

(12) $$s=\frac{1}{2{\left({3\pi }^{2}\right)}^{\frac{1}{3}}}\frac{\left|\nabla \rho \right|}{\rho^{\frac{4}{3}}}.$$

For values of *ρ* close to zero, s tends to diverge, typically in intra- or intermolecular regions. To characterize the type of interaction at these critical points, a real space function, ${sign(\lambda }_{2})\rho$, is used [32]. Isosurfaces for RDG can also be drawn, which allows visualizing the different types of non-covalent interactions. RDG analysis has been applied to help categorize non-covalent interactions in different systems [31,55]. RDG properties were computed using the wavefunction analysis free program Multiwfn [56]. The draw of the isosurfaces and molecules for both QTAIM and RDG analysis was made with VMD software version 1.9.3 [57]. To ensure that the NCIs would be well analyzed, the fullerenes dimers were fully optimized at the equilibrium distance obtained from the PEC calculations. 

## 4. Conclusions

The present work was devoted to obtaining the spectroscopic properties and characterizing the intermolecular potential and hence, the stability of the fullerene dimers ${\left(C_{20}\right)}_{2},$ ${\left(C_{24}\right)}_{2}$, ${\left(C_{36}\right)}_{2}$, ${\left(C_{60}\right)}_{2}$, ${\left(C_{70}\right)}_{2}$, and ${\left(C_{84}\right)}_{2}$ by means of the solution of the Nuclear Schrodinger Equation. The fitting procedure of the PECs obtained proved to be satisfactory, resulting in errors smaller than 0.05 kcal mol^−1^ in the equilibrium region. The interaction between the buckyballs increases with the system size. Lifetime calculations showed that the studied dimers are stable for more than 1 ps, which increases accordingly with the number of carbon atoms. 

Both Dunham’s and DVR’s methods returned similar results, ensuring the reliability of the methodology. The spectroscopic constants $\omega_{e},$ $\omega_{e}x_{e}$ and $B_{e}$ were determined and revealed that the anharmonicity effects associated with the *ω_e_x_e_* constant become more operative for the smallest dimers representing one-tenth of the harmonic frequency for the (*C*_20_)_2_ and (*C*_24_)_2_ dimers. The vibrational population for each state and the temperature effects of the Boltzmann distribution of the quantized levels were calculated. At room temperature (300K), less than 25% of the dimers lie on the vibrational ground state, which indicates that, for a good description of the normal modes, the inclusion of higher excited states is mandatory. On the other hand, at a temperature of ~10 K, almost 100% of the dimers are in the ground state.

QTAIM and RDG analyses indicate that the fullerenes dimers are stabilized due to van der Waals interactions. Investigation of the bond path and BCP descriptors enabled the determination of the preferential interaction configuration of the (*C*_60_)_2_ dimer and compared it with experimental results obtained with *C*_60_ in-bulk. We expect that the theoretical data provided here should be useful for future studies and dimers applications in different areas. 

## Figures and Tables

**Table 1:** Equilibrium distance *R_e_* and dissociation energy *D_e_* obtained in this work for ${\left(C_{20}\right)}_{2}$ , ${\left(C_{24}\right)}_{2}$ , ${\left(C_{36}\right)}_{2}$ , ${\left(C_{60}\right)}_{2}$ , ${\left(C_{70}\right)}_{2}$ and ${\left(C_{84}\right)}_{2}$ dimers.

Fullerene Dimers	$R_{e}\left(Å\right)$	$D_{e}$
${\left(C_{20}\right)}_{2}$	7.657	1.399
${\left(C_{24}\right)}_{2}$	7.690	2.905
${\left(C_{36}\right)}_{2}$	8.143	4.523
${\left(C_{60}\right)}_{2}$	10.069	6.580
	9.1 [24] *	(6.342 ± 1.845) [25] *
	10.05 [25,26,27] *	6.389 [27] *
	10.0 [28] *	7.748 [28] *
${\left(C_{70}\right)}_{2}$	10.201	7.667
	10.110 [25] *	(7.218 ± 1.845) [26] *
${\left(C_{84}\right)}_{2}$	10.467	10.414

* Reference [24] had the values estimated for covalently bonded dimers, [25,26] for free dimers, [27,28] obtained values for dimers in a cluster of fullerenes.

**Table 2:** Spectroscopic constants (in ${cm}^{-1}$) calculated by Dunham’s and DVR methods for ${\left(C_{20}\right)}_{2}$, ${\left(C_{24}\right)}_{2}$, ${\left(C_{36}\right)}_{2}$, ${\left(C_{60}\right)}_{2}$, ${\left(C_{70}\right)}_{2}$, and ${\left(C_{84}\right)}_{2}$ dimers.

System	Method	$B_{e}\times 10^{-4}$	$\omega_{e}$	${\omega_{e}x}_{e}\times 10^{-2}$	$\alpha_{e}\times 10^{-7}$
${\left(C_{20}\right)}_{2}$	Dunham	23.94	20.77	26.60	157.59
	DVR	-	20.76	26.82	157.45
${\left(C_{24}\right)}_{2}$	Dunham	19.78	29.91	24.49	74.52
	DVR	-	29.19	22.37	76.05
${\left(C_{36}\right)}_{2}$	Dunham	11.76	29.39	15.45	28.30
	DVR	-	29.38	15.46	29.82
${\left(C_{60}\right)}_{2}$	Dunham	4.61	27.33	9.29	6.00
	DVR	-	27.28	9.18	3.00
${\left(C_{70}\right)}_{2}$	Dunham	3.85	26.33	7.54	4.25
	DVR	-	26.32	7.55	6.25
${\left(C_{84}\right)}_{2}$	Dunham	3.05	29.04	6.90	3.05
	DVR	-	29.89	7.64	2.26

**Table 3:** Quantized energies of the first ten vibrational levels (in cm^−1^) for the ${\left(C_{20}\right)}_{2}$, ${\left(C_{24}\right)}_{2}$, ${\left(C_{36}\right)}_{2}$, ${\left(C_{60}\right)}_{2}$, ${\left(C_{70}\right)}_{2}$, and ${\left(C_{84}\right)}_{2}$ dimers.

Υ	${\left(C_{20}\right)}_{2}$	${\left(C_{24}\right)}_{2}$	${\left(C_{36}\right)}_{2}$	${\left(C_{60}\right)}_{2}$	${\left(C_{70}\right)}_{2}$	${\left(C_{84}\right)}_{2}$
0	10.3	14.8	14.5	13.6	13.1	19.1
1	30.0	43.5	43.6	40.7	39.3	44.7
2	50.0	71.8	72.4	67.6	65.3	74.3
3	69.0	99.7	100.8	94.3	91.2	103.7
4	87.98	127.0	128.9	120.8	116.9	133.0
5	106.1	154.0	156.8	147.2	142.5	162.1
6	123.6	180.4	184.3	173.4	167.9	191.1
7	140.7	206.4	211.5	199.4	193.2	219.9
8	157.2	231.9	238.4	225.2	218.3	248.6
9	173.2	256.9	254.5	250.7	243.2	277.1

## Data Availability

The data presented in this study are available in the article and the Supplementary Material.

## Supplementary Materials

The following supporting information can be downloaded at: https://www.mdpi.com/article/10.3390/molecules28135023/s1, Figure S1: Potential energy curves (top) fitted with analytic Rydberg function for the (C20)2 dimer calculated at ωB97xD/6−31G(d) level of theory. Point-by-point error (bottom) is also plotted to magnify the overall fitting accuracy in the entire range of internuclear separation.; Figure S2: Potential energy curves (top) fitted with analytic Rydberg function for the (C24)2 dimer calculated at ωB97xD/6−31G(d) level of theory. Point-by-point error (bottom) is also plotted to magnify the overall fitting accuracy in the entire range of internuclear separation.; Figure S3: Potential energy curves (top) fitted with analytic Rydberg function for the (C36)2 dimer calculated at ωB97xD/6−31G(d) level of theory. Point-by-point error (bottom) is also plotted to magnify the overall fitting accuracy in the entire range of internuclear separation.; Figure S4: Potential energy curves (top) fitted with analytic Rydberg function for the (C60)2 dimer calculated at ωB97xD/6−31G(d) level of theory. Point-by-point error (bottom) is also plotted to magnify the overall fitting accuracy in the entire range of internuclear separation.; Figure S5: Potential energy curves (top) fitted with analytic Rydberg function for the (C70)2 dimer calculated at ωB97xD/6−31G(d) level of theory. Point-by-point error (bottom) is also plotted to magnify the overall fitting accuracy in the entire range of internuclear separation.; Figure S6: Potential energy curves (top) fitted with analytic Rydberg function for the (C84)2 dimer calculated at ωB97xD/6−31G(d) level of theory. Point-by-point error (bottom) is also plotted to magnify the overall fitting accuracy in the entire range of internuclear separation.; Figure S7: (A) Optimized C60-fullerene dimer as determined from ωB97xD/6−31G(d) calculation depicting the most stable orientation, where the pentagon of C60 faces the inter-pentagon bond of the adjacent monomer. (B) side view and (C) superimposed view to highlight the relative orientation between the monomers.; Table S1: Parameters utilized for the potential fit through the Rydberg function for the (C20)2, (C24)2, and (C36)2 dimers.; Table S2: Parameters utilized for the potential fit through the Rydberg function for the (C60)2, (C70)2, and (C84)2 dimers.; Table S3: Quantized energies of the first ten vibrational levels (in cm−1) for the (C20)2, (C24)2, (C36)2 (C60)2, (C70)2, and (C84)2 dimers; Table S4: Lifetime values for the (C20)2, (C24)2, (C36)2 (C60)2, (C70)2 and (C84)2 dimers from a temperature of 200 K until 500 K; Table S5: Coefficients of fitted expressions of the ωB97XD/6−31G(d)-derived intermolecular potential for the (C60)2 non-covalent dimer. Fitting parameters for the Ryd6 analytical potential are shown in Table S2. Table S6: Electron density ρcp (in e/a03), the Laplacian of the electron density ∇2pcp (in e/a05), and the total energy HBCP (in Hartree) associated with the bond-critical point occurring along the bond path connecting each monomer for the (C20)2, (C24)2, (C36)2, (C60)2, (C70)2, and (C84)2 dimers, separated by a distance Re. Table S7: Coordinates for the C20, C24, C36, C60, C70, and C84 optimized fullerenes; Table S8: Coordinates for the (C20)2, (C24)2, (*C*_36_)_2_ (*C*_60_)_2_, (*C*_70_)_2,_ and (*C*_84_)_2_ dimers at the equilibrium point. References [58,59,60] are cited in the supplementary materials.
