# Supplementary material for: (Ro)vibrational Spectroscopic Constants, Lifetime and QTAIM Evaluation of Fullerene Dimers Stability
Source: Molecules. 2023 Jun 27;28(13):5023. doi: 10.3390/molecules28135023 (PMC10343245; doi:10.3390/molecules28135023)
Supplement: Supplementary file 1 [file molecules-28-05023-s001.zip › molecules-2404467-supplementary.pdf]

(Ro)vibrational spectroscopic constants, lifetime and QTAIM evaluation of fullerene dimers stability

Rodrigo A. Lemos Silva,[1,3] Mateus R. Barbosa,[5] Caio R. Martins[3], Daniel F. Scalabrini Machado,[4] Luciano Ribeiro,[3,*] Heibbe C. B. de Oliveira[5,*] and Demétrio A. da Silva Filho[2]

[1] Instituto Federal de Educação, Ciência e Tecnologia de Goiás (IFG), Câmpus Jataí, CP 75804-714, Jataí, GO, Brazil

[2] Grupo de Semicondutores Orgânicos, Instituto de Física, Campus Darcy Ribeiro, Universidade de Brasília, CP 4478, 70919-970, Brasília, DF, Brazil

[3] Grupo de Química Teórica e Estrutural de Anápolis (GQTEA), Campus Central Anápolis de Ciências Exatas e Tecnológicas Henrique Santillo, Universidade Estadual de Goiás, CP 459, Anápolis, GO, Brazil

[4] Laboratório de Modelagem Molecular de Sistemas Complexos (LMSC), Instituto de Química, Universidade de Brasília, CP 4478, 70919-970, Brasília, DF, Brazil

[5] Laboratório de Estrutura Eletrônica e Dinâmica Molecular (LEEDMOL), Instituto de Química, Universidade Federal de Goiás, CP 131, Goiânia, GO 74001-970, Brazil

* lribeiro@ueg.br; * heibbe@ufg.br

Supporting Information

Contents

Table S1……………………………………………………………………………2
Table S2……………………………………………………………………………2
Table S3……………………………………………………………………………3
Table S4……………………………………………………………………………3
Table S5……………………………………………………………………………4
Table S6……………………………………………………………………………5
Table S7……………………………………………………………………………13
Table S8……………………………………………………………………………19
Figure S1……………………………………………………………………………5
Figure S2……………………………………………………………………………6
Figure S3……………………………………………………………………………7
Figure S4……………………………………………………………………………8
Figure S5……………………………………………………………………………9
Figure S6……………………………………………………………………………10
Figure S7……………………………………………………………………………13

Table S1. Parameters utilized for the potential fit through the Rydberg function for the $(C_{20})_2$, $(C_{24})_2$ e $(C_{36})_2$ dimers.

Parameters	$(C_{20})_2$	$(C_{24})_2$	$(C_{36})_2$
a_1(Å^{-1})	1.382296283748	1.655520259343	1.489512569107
a_2(Å^{-2})	-0.613336853427	-0.511033188834	-0.640266331647
a_3(Å^{-3})	0.431444235886	0.211579334300	0.314330181242
a_4(Å^{-4})	-0.081274263814	0.232102580314	0.095042385956
a_5(Å^{-5})	0.008407447218	-0.096380917163	-0.048715598849
a_6(Å^{-6})	0.000686843085	0.015090390276	0.007341205437
D_e(kcal/mol)	1.399500015242	2.905001534972	4.522900105120
R_0(Å)	7.657192031840	7.690292985391	8.142999649003
χ^2(kcal/mol)	3.289794795301E-05	4.693895980794E-05	9.559571764342E-05

Table S2. Parameters utilized for the potential fit through the Rydberg function for the $(C_{60})_2$, $(C_{70})_2$ e $(C_{84})_2$ dimers.

Parameters	$(C_{60})_2$	$(C_{70})_2$	$(C_{84})_2$
a_1(Å^{-1})	1.405307280248	1.351086576379	1.510531912516
a_2(Å^{-2})	-0.745280808494	-0.738948458009	-0.590635149516
a_3(Å^{-3})	0.407860812683	0.400421160872	0.372092153230
a_4(Å^{-4})	0.004071309685	0.003805208695	0.067208088029
a_5(Å^{-5})	-0.020584302212	-0.020697907717	-0.042140891776
a_6(Å^{-6})	0.003826456070	0.003541573910	0.007565856445
D_e(kcal/mol)	6.580230974750	7.667389935915	10.413984425274
R_0(Å)	10.068927683590	10.201000211979	10.467438271011
χ^2(kcal/mol)	8.546616880279E-05	2.36163185089E-04	1.584479030689E-4

Table S3. Quantized energies of the first ten vibrational levels (in cm^{-1}) for the $(C_{20})_2$, $(C_{24})_2$, $(C_{36})_2$, $(C_{60})_2$, $(C_{70})_2$ and $(C_{84})_2$ dimers.

ν	$(C_{20})_2$	$(C_{24})_2$	$(C_{36})_2$	$(C_{60})_2$	$(C_{70})_2$	$(C_{84})_2$
0	10.3	14.8	14.5	13.6	13.1	14.9
1	30.5	43.5	43.6	40.7	39.3	44.7
2	50.2	71.8	72.4	67.6	65.3	74.3
3	69.3	99.7	100.8	94.3	91.2	103.7
4	88.1	127.0	128.9	120.8	116.9	133.0
5	106.1	154.0	156.8	147.2	142.5	162.1
6	123.6	180.4	184.3	173.4	167.9	191.1
7	140.7	206.4	211.5	199.4	193.2	219.9
8	157.2	231.9	238.4	225.2	218.3	248.6
9	173.2	256.9	254.5	250.7	243.2	277.1

Table S4. Lifetime values for the $(C_{20})_2$, $(C_{24})_2$, $(C_{36})_2$, $(C_{60})_2$, $(C_{70})_2$ and $(C_{84})_2$ dimers from temperature of 200 K until 500 K.

Temperature (K)	Life time (s)					
	$(C_{20})_2$	$(C_{24})_2$	$(C_{36})_2$	$(C_{60})_2$	$(C_{70})_2$	$(C_{84})_2$
200	5.047E-11	1.535E-09	8.960E-08	1.718E-05	2.721E-04	2.476E-01
210	4.283E-11	1.090E-09	5.237E-08	7.847E-06	1.090E-04	7.146E-02
220	3.689E-11	7.977E-10	3.214E-08	3.848E-06	4.750E-05	2.309E-02
230	3.219E-11	6.001E-10	2.058E-08	2.008E-06	2.224E-05	8.231E-03
240	2.841E-11	4.622E-10	1.368E-08	1.106E-06	1.109E-05	3.198E-03
250	2.533E-11	3.636E-10	9.394E-09	6.389E-07	5.849E-06	1.340E-03
260	2.278E-11	2.913E-10	6.640E-09	3.850E-07	3.240E-06	6.002E-04
270	2.065E-11	2.372E-10	4.816E-09	2.409E-07	1.875E-06	2.854E-04
280	1.885E-11	1.961E-10	3.574E-09	1.559E-07	1.128E-06	1.431E-04
290	1.731E-11	1.642E-10	2.707E-09	1.039E-07	7.032E-07	7.523E-05
300	1.599E-11	1.391E-10	2.089E-09	7.117E-08	4.523E-07	4.129E-05
310	1.485E-11	1.192E-10	1.639E-09	4.996E-08	2.993E-07	2.356E-05
320	1.385E-11	1.031E-10	1.306E-09	3.585E-08	2.032E-07	1.392E-05
330	1.298E-11	8.992E-11	1.055E-09	2.625E-08	1.413E-07	8.492E-06
340	1.220E-11	7.909E-11	8.626E-10	1.957E-08	1.003E-07	5.333E-06
350	1.152E-11	7.007E-11	7.137E-10	1.484E-08	7.267E-08	3.439E-06
360	1.090E-11	6.250E-11	5.967E-10	1.143E-08	5.358E-08	2.273E-06
370	1.035E-11	5.609E-11	5.038E-10	8.927E-09	4.016E-08	1.536E-06

380	9.858E-12	5.063E-11	4.291E-10	7.064E-09	3.056E-08	1.060E-06
390	9.410E-12	4.594E-11	3.685E-10	5.657E-09	2.359E-08	7.451E-07
400	9.003E-12	4.189E-11	3.189E-10	4.581E-09	1.844E-08	5.333E-07
410	8.632E-12	3.837E-11	2.780E-10	3.748E-09	1.459E-08	3.879E-07
420	8.294E-12	3.529E-11	2.438E-10	3.095E-09	1.168E-08	2.865E-07
430	7.983E-12	3.258E-11	2.152E-10	2.580E-09	9.439E-09	2.146E-07
440	7.697E-12	3.019E-11	1.910E-10	2.168E-09	7.706E-09	1.628E-07
450	7.434E-12	2.807E-11	1.705E-10	1.836E-09	6.347E-09	1.251E-07
460	7.190E-12	2.619E-11	1.529E-10	1.566E-09	5.273E-09	9.723E-08
470	6.965E-12	2.450E-11	1.377E-10	1.345E-09	4.415E-09	7.637E-08
480	6.755E-12	2.298E-11	1.246E-10	1.162E-09	3.724E-09	6.060E-08
490	6.560E-12	2.162E-11	1.132E-10	1.010E-09	3.163E-09	4.854E-08
500	6.378E-12	2.038E-11	1.033E-10	8.833E-10	2.704E-09	3.923E-08

Table S5. Coefficients of fitted expressions of the ωB97XD/6−31G(d)-derived intermolecular potential for the $(C_{60})_2$ non-covalent dimer. Fitting parameters for the Ryd6 analytical potential are shown in Tab. S2.

Girifalco Potential [1]

$$U(R) = -\frac{N^2 A}{12(2r)^6}\left(\frac{1}{s(s-1)^3} + \frac{1}{s(s+1)^3} - \frac{2}{s^4}\right) + \frac{N^2 B}{90(2r)^{12}}\left(\frac{1}{s(s-1)^9} + \frac{1}{s(s+1)^9} - \frac{2}{s^{10}}\right),$$

with, $s = -\frac{R}{2r}$, and r is the radius of the fullerenes, taken to be 3.55Å by Girifalco and $N = 60$ is the number of carbon atoms of the fullerene.

$A(Å^6 \cdot kcal \cdot mol^{-1})$	$B(Å^{12} \cdot kcal \cdot mol^{-1})$		$\bar{R}^2$
420.80178	617489.63712		0.98358

Smith-Thakkar Potential [2]

$$U(R) = \frac{A}{(R^2 - d^2)^6} - \frac{B}{(R^2 - d^2)^3}$$

$A(Å^{12} \cdot kcal \cdot mol^{-1})$	$B(Å^6 \cdot kcal \cdot mol^{-1})$	$d(Å)$	$\bar{R}^2$
7.96778×10^{10}	1.4149×10^6	7.24689	0.99577

Lim Potential [3]

$$U(R) = D\left[\frac{n}{m-n}\left(\frac{L}{R}\right)^{\frac{m}{2}} e^{\frac{m}{2}\left(1-\frac{R}{L}\right)} - \frac{m}{m-n}\left(\frac{L}{R}\right)^{\frac{n}{2}} e^{\frac{n}{2}\left(1-\frac{R}{L}\right)}\right]$$

$D(kcal \cdot mol^{-1})$	m and n	$L(Å)$	$\bar{R}^2$
6.36667	33.8038 and 9.1112	10.06369	0.99725

Table S6. Electron density ρ_{cp} (in e/a_0^3), the Laplacian of the electron density $\nabla^2\rho_{cp}$ (in e/a_0^5) and the total energy H_{BCP} (in Hartree) associated with the bond critical point occurring along the bond path connecting each monomer for the $(C_{20})_2$, $(C_{24})_2$, $(C_{36})_2$, $(C_{60})_2$, $(C_{70})_2$ and $(C_{84})_2$ dimers, separated by a distance R_e.

**Dimers**	QTAIM topological parameters				
	CP	Index	$\rho_{CP} \times 10^{-3}$	$\nabla^2\rho_{CP} \times 10^{-2}$	$H_{CP} \times 10^{-4}$
$(C_{20})_2$	BCP	1	4.4568	1.2563	6.3101
	BCP	2	6.2001	1.8118	8.9895
	BCP	3	5.8758	1.5980	7.4308
	RCP	4	3.7311	1.0302	5.0755
	RCP	5	3.5599	1.2066	5.9805
	RCP	6	4.3480	1.2725	6.3435
	RCP	7	5.5169	1.5654	7.5710
	CCP	8	3.5296	1.1103	5.3195
	CCP	9	3.5563	1.2515	6.1566
$(C_{24})_2$	BCP	1	5.3661	1.5663	7.9016
	BCP	2	8.0237	2.3419	1.1115
	RCP	3	4.4836	1.3144	6.3568
	RCP	4	4.8776	1.3915	6.6206
	CCP	5	3.7929	1.3281	6.1190
$(C_{36})_2$	BCP	1	5.7331	1.5779	7.3033
	BCP	2	6.0909	1.8813	9.4612
	BCP	3	5.7072	1.5697	7.2770
	RCP	4	5.2735	1.5049	7.0835
	RCP	5	4.4032	1.3160	6.2448
	RCP	6	4.1315	1.2490	5.8825

	RCP	7	5.4473	1.5517	7.3004
	CCP	8	4.0323	1.3305	5.8395
	CCP	9	3.9658	1.2994	5.7515
$(C_{60})_2$	BCP	1	6.6077	2.0247	1.0074
	BCP	2	6.6040	2.0235	1.0068
	RCP	3	5.2660	1.7879	8.4140
$(C_{70})_2$	BCP	1	5.5690	1.5873	7.5764
	BCP	2	5.5388	1.5748	7.5083
	BCP	3	5.5690	1.5873	7.5764
	BCP	4	5.5388	1.5748	7.5083
	RCP	5	5.4401	1.4766	6.8657
	RCP	6	3.8613	1.1319	5.5652
	RCP	7	3.8092	1.1181	5.5068
	RCP	8	5.4401	1.4766	6.8657
	CCP	9	1.8620	8.8365	4.4566
$(C_{84})_2$	BCP	1	5.4192	1.6214	8.1633
	BCP	2	5.4316	1.6254	8.1823
	BCP	3	5.4306	1.6251	8.1798
	BCP	4	5.4583	1.6339	8.2199
	BCP	5	5.4567	1.6334	8.2188
	BCP	6	5.4726	1.6382	8.2399
	RCP	7	4.9567	1.4543	7.2929
	RCP	8	4.9552	1.4544	7.2948
	RCP	9	4.9735	1.4601	7.3244

	RCP	10	4.9724	1.4597	7.3225
	RCP	11	4.9923	1.4658	7.3538
	RCP	12	4.9925	1.4654	7.3503

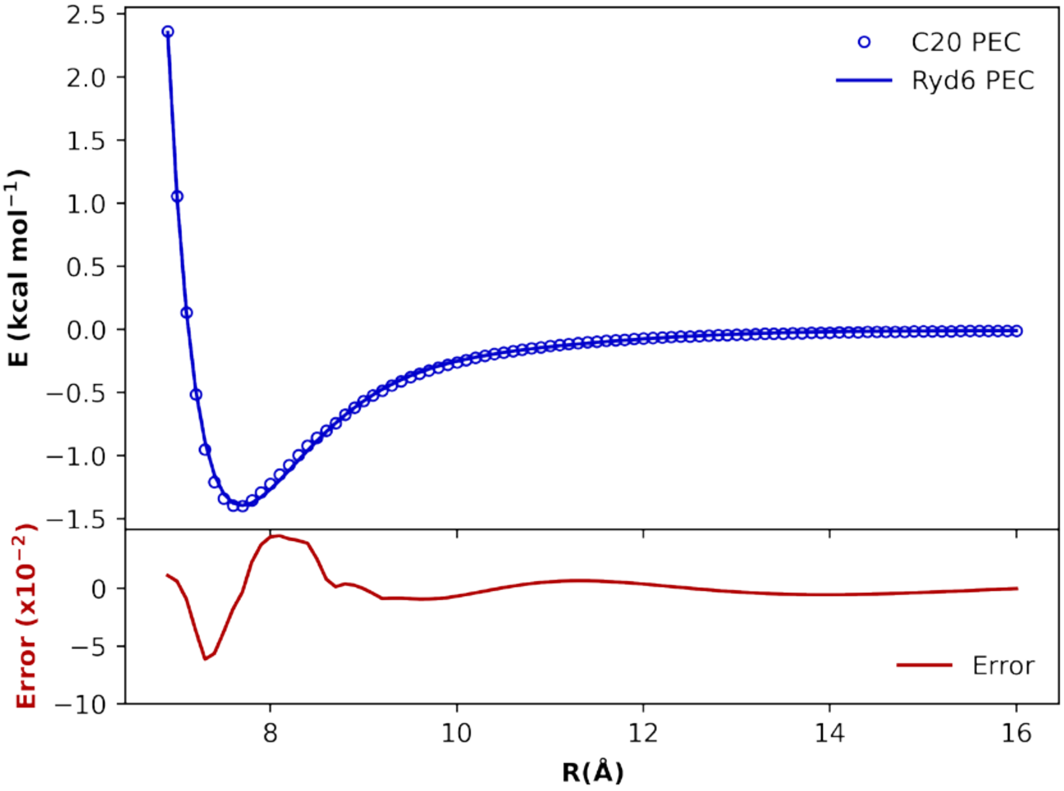

Figure S1. Potential energy curves (top) fitted with analytic Rydberg function for the $(C_{20})_2$ dimer calculated at ωB97xD/6−31G(d) level of theory. Point by point error (bottom) is also plotted to magnify the overall fitting accuracy in the entire range of internuclear separation.

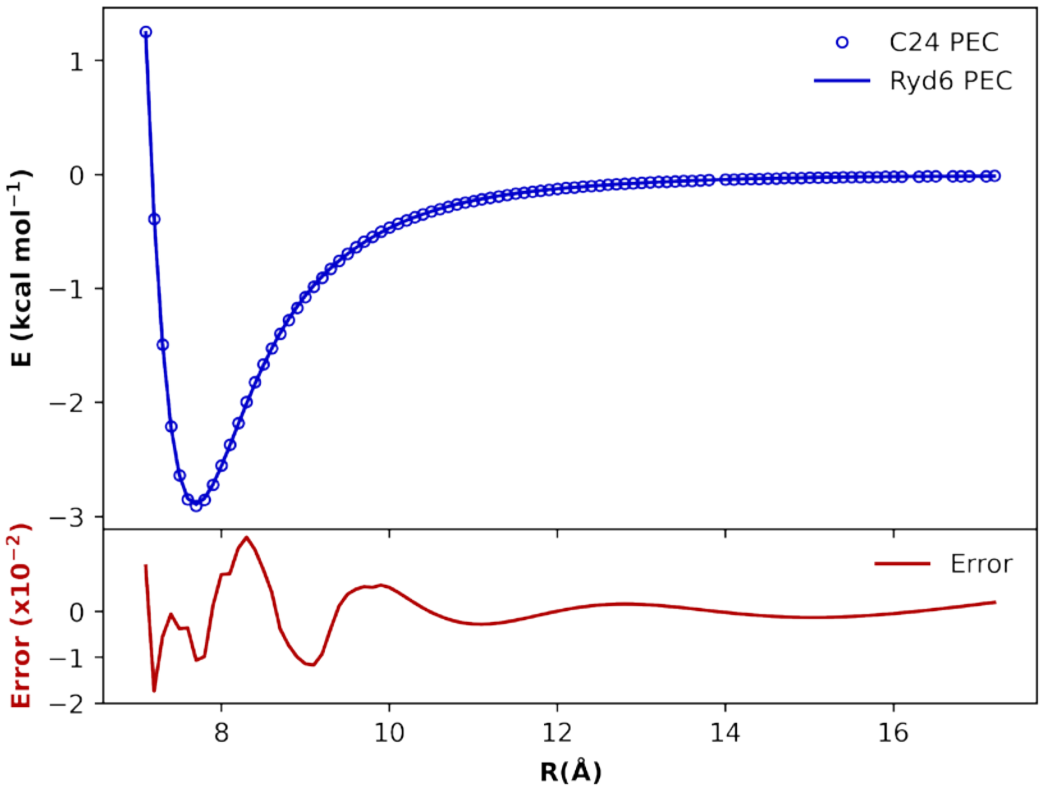

Figure S2. Potential energy curves (top) fitted with analytic Rydberg function for the $(C_{24})_2$ dimer calculated at ωB97xD/6−31G(d) level of theory. Point by point error (bottom) is also plotted to magnify the overall fitting accuracy in the entire range of internuclear separation.

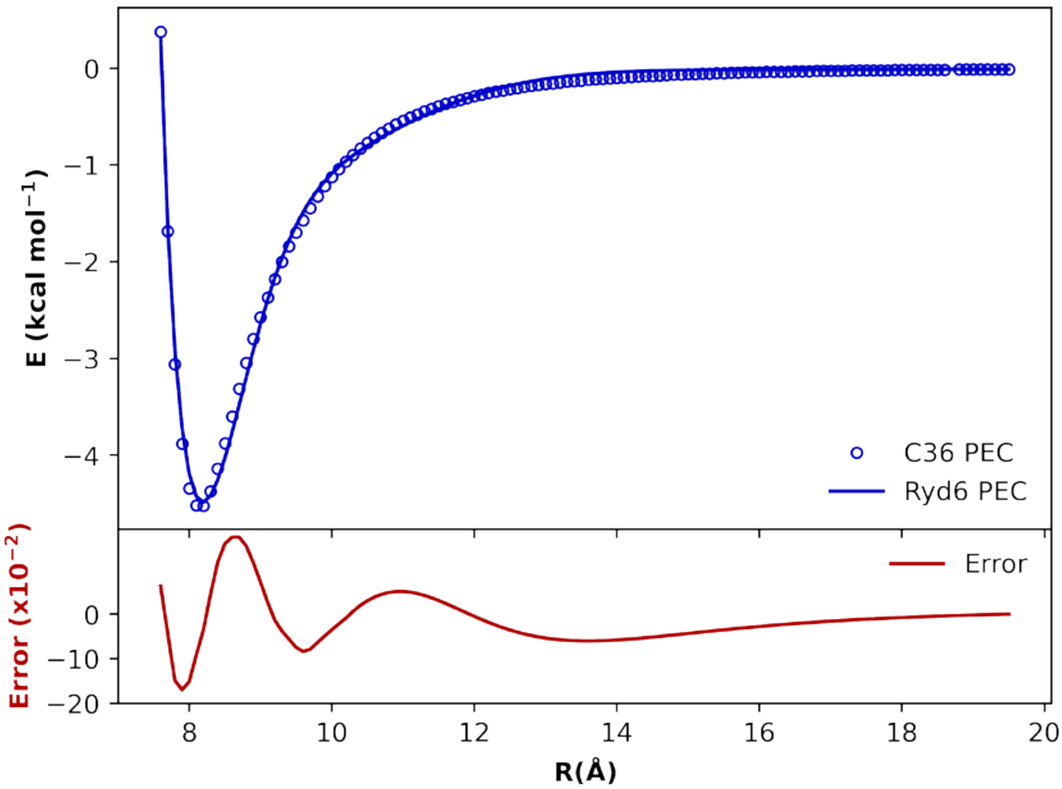

Figure S3. Potential energy curves (top) fitted with analytic Rydberg function for the $(C_{36})_2$ dimer calculated at ωB97xD/6−31G(d) level of theory. Point by point error (bottom) is also plotted to magnify the overall fitting accuracy in the entire range of internuclear separation.

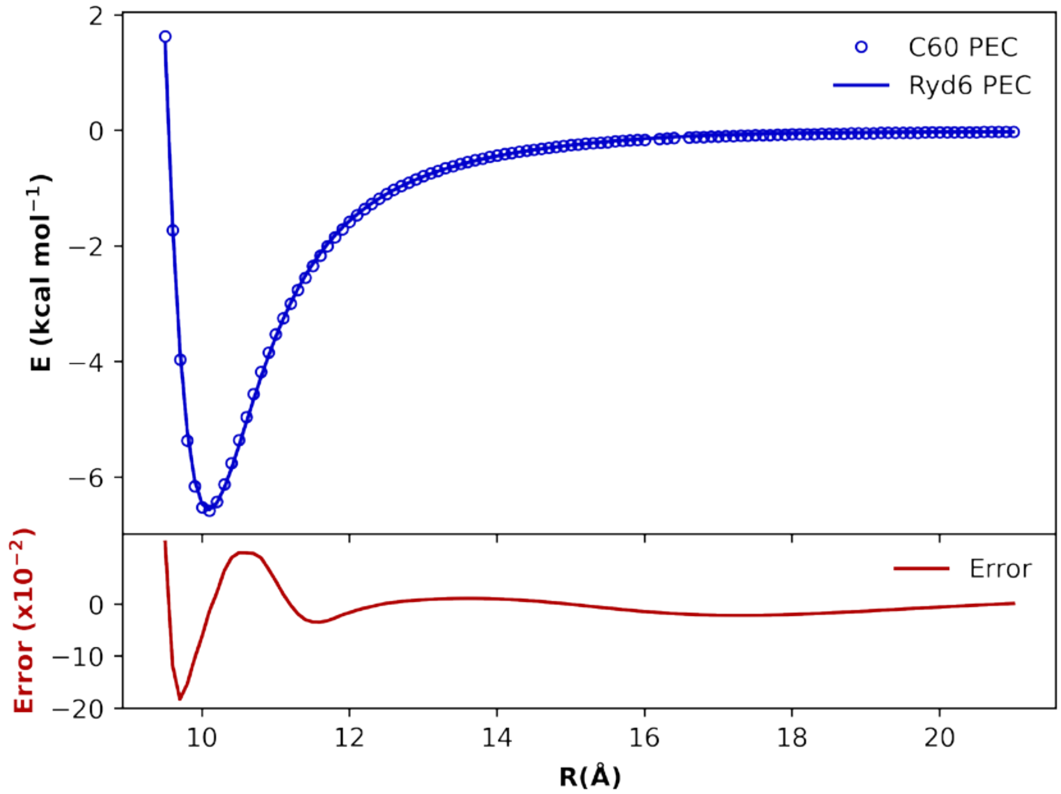

Figure S4. Potential energy curves (top) fitted with analytic Rydberg function for the $(C_{60})_2$ dimer calculated at ωB97xD/6−31G(d) level of theory. Point by point error (bottom) is also plotted to magnify the overall fitting accuracy in the entire range of internuclear separation.

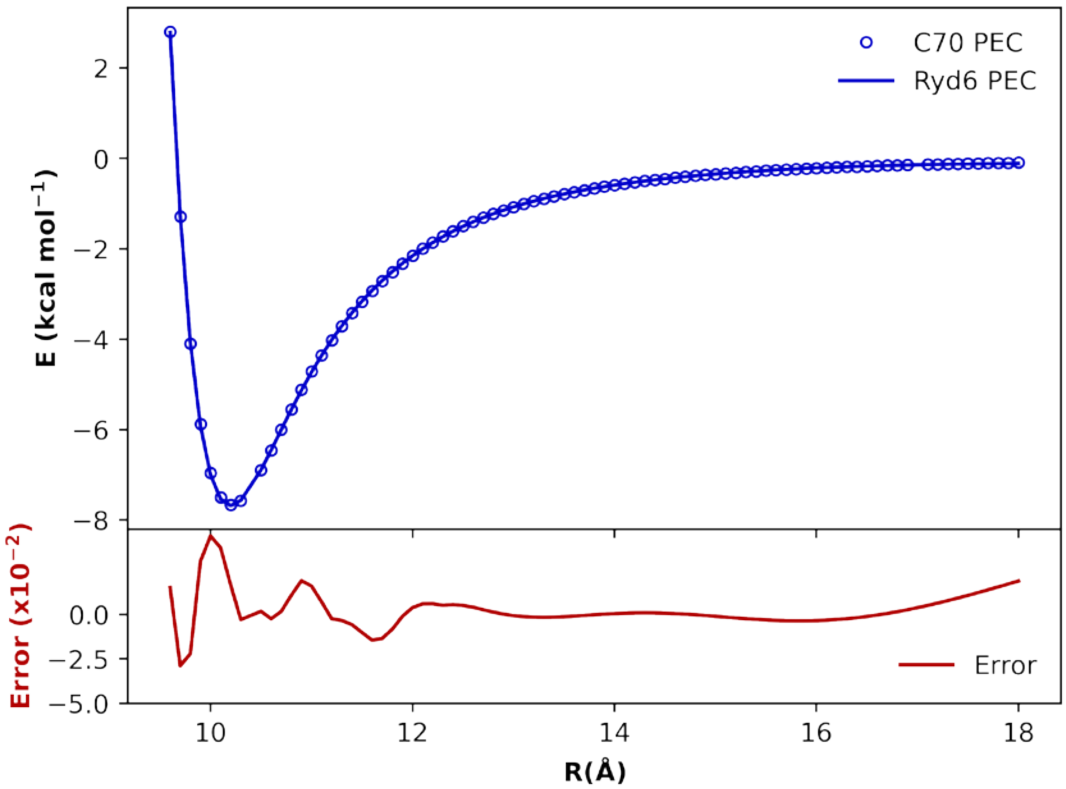

Figure S5. Potential energy curves (top) fitted with analytic Rydberg function for the $(C_{70})_2$ dimer calculated at ωB97xD/6−31G(d) level of theory. Point by point error (bottom) is also plotted to magnify the overall fitting accuracy in the entire range of internuclear separation.

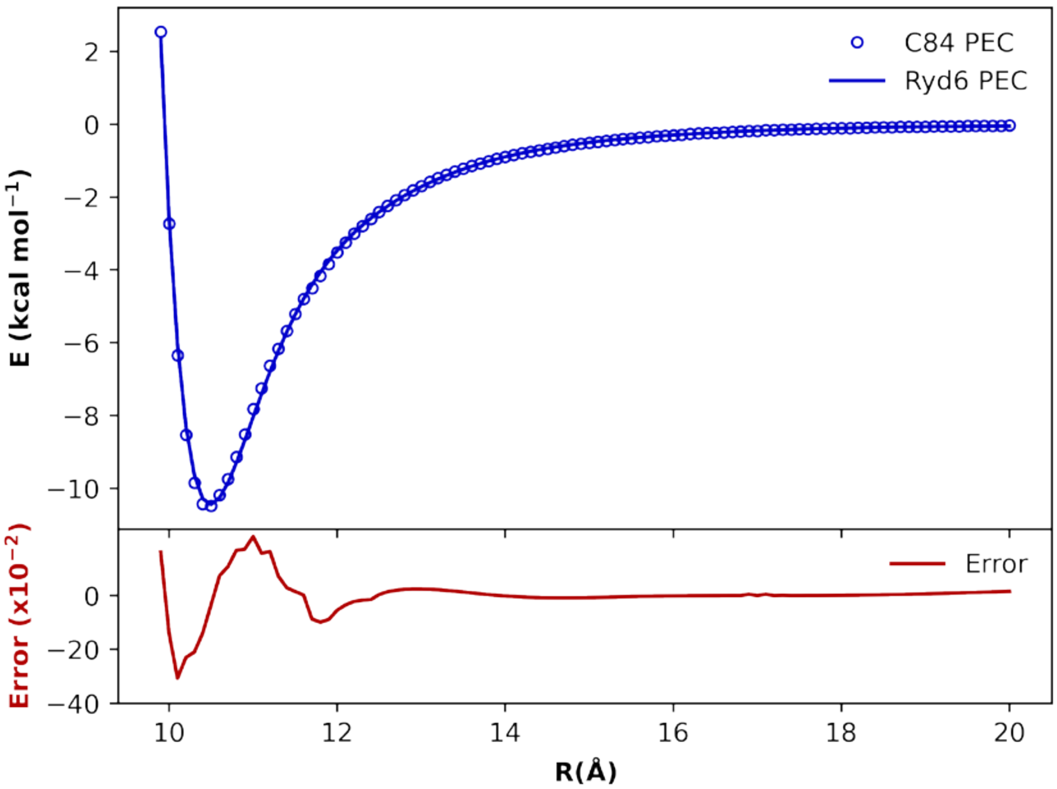

Figure S6. Potential energy curves (top) fitted with analytic Rydberg function for the $(C_{84})_2$ dimer calculated at ωB97xD/6−31G(d) level of theory. Point by point error (bottom) is also plotted to magnify the overall fitting accuracy in the entire range of internuclear separation.

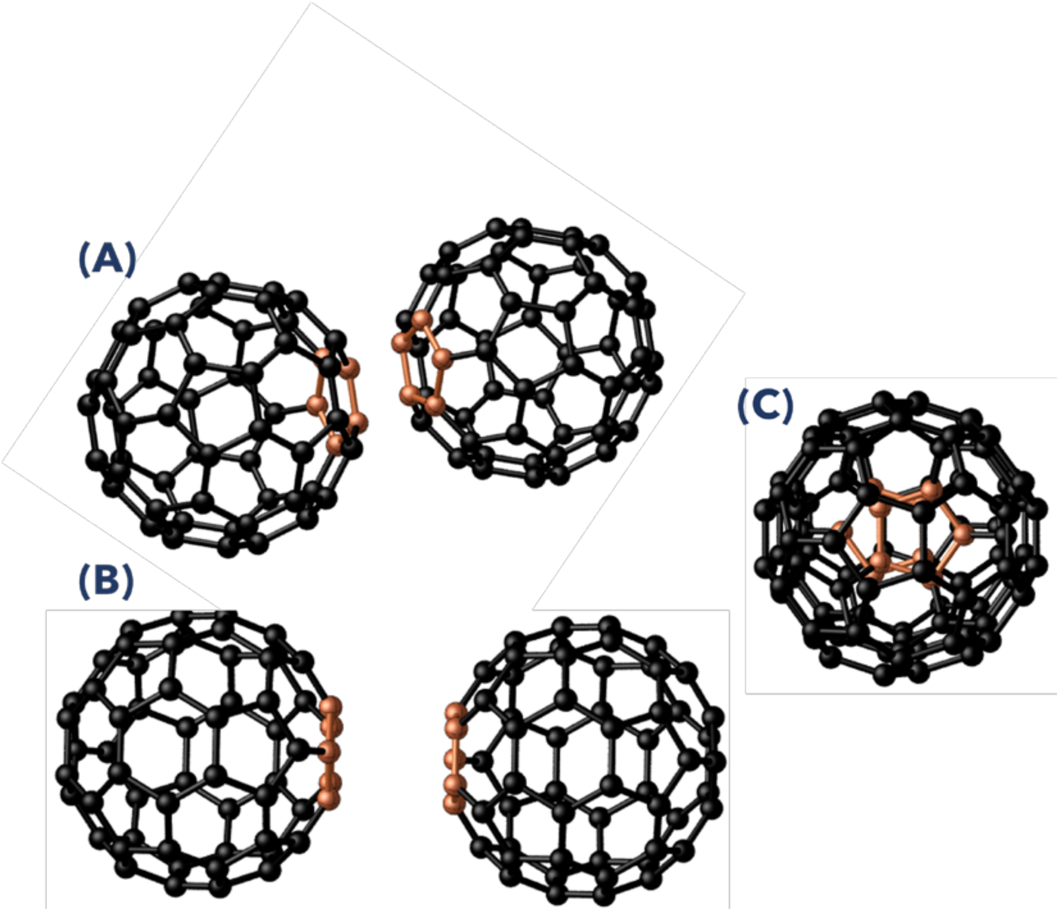

Figure S7. (A) Optimized C_{60}-fullerene dimer as determined from ωB97xD/6−31G(d) calculation depicting the most stable orientation, where the pentagon of C_{60} faces the inter-pentagon bond of the adjacent monomer. (B) side view and (C) superimposed view to highlight the relative orientation between the monomers.

Table S7. Coordinates for the C_{20}, C_{24}, C_{36} , C_{60}, C_{70} and C_{84} optimized fullerenes.

C_{20}			
C	-1.17424900	1.18813100	-1.10398800
C	0.00011000	0.73578000	-1.80070600
C	-0.76566400	1.94690900	0.01685100
C	0.76603500	1.94677200	0.01688500
C	1.17452200	1.18791000	-1.10393600
C	-1.17375900	1.16564000	1.12382200
C	0.00002000	0.70121300	1.81334500
C	1.17392500	1.16542200	1.12387200
C	-1.96801900	-0.00628000	0.69593200
C	-1.17452300	-1.18791200	1.10393600
C	-0.00011100	-0.73578000	1.80070600
C	-1.96797800	0.00664300	-0.69601100
C	-1.17392600	-1.16542200	-1.12387300

C	-0.76603400	-1.94677000	-0.01688500
C	-0.00002000	-0.70121300	-1.81334300
C	1.17376100	-1.16564000	-1.12382200
C	0.76566400	-1.94690700	-0.01685100
C	1.17424900	-1.18813100	1.10398900
C	1.96797700	-0.00664300	0.69601100
C	1.96802000	0.00627900	-0.69593300
C_{24}			
C	-1.81487400	-1.23980200	1.01859700
C	-1.86280400	0.05950500	1.68481100
C	-2.96293000	-1.90865000	1.28189700
C	-3.28151700	0.24757500	2.21608500
C	-3.95335300	-0.97533900	1.97367000
C	-3.56581600	-2.32560600	0.01822000
C	-4.85757500	-1.91878600	-0.00513200
C	-5.12578100	-0.99397900	1.17937200
C	-2.79162100	-1.64004900	-1.10469100
C	-1.70481200	-0.97950100	-0.48146400
C	-1.55206600	0.42128000	-0.62332900
C	-3.72549200	-0.89981300	-1.86984200
C	-3.57266400	0.50100000	-2.01178200
C	-2.48606800	1.16158300	-1.38853100
C	-5.08633200	-1.14544000	-1.22326900
C	-2.65727700	2.01735200	0.80092500
C	-1.58422500	1.02624000	0.77795200
C	-3.10484800	2.20651700	-0.46344400
C	-4.55192000	2.00719000	-0.48615800
C	-4.85577200	1.12068900	-1.46416600
C	-5.65197000	0.03911200	-0.88921300
C	-3.78219900	1.45187600	1.66415000
C	-4.95462000	1.43338000	0.86991100
C	-5.62639900	0.21042500	0.62747100
C_{36}			
C	-1.41065800	-1.72915300	1.50394700
C	1.41077000	-1.72956800	1.50422500
C	0.72002500	-2.42201600	0.47118800
C	-0.71996400	-2.42211800	0.47119900
C	-2.60358100	-1.05914200	0.92120200
C	-2.60379200	0.26797600	1.37752900
C	-2.60389100	1.32709500	0.45638500
C	-2.60380800	-1.32712800	-0.45635400
C	-2.60388600	-0.26801700	-1.37749200
C	-2.60354200	1.05899400	-0.92106000
C	0.71995800	-1.61909600	-1.86173900
C	-0.71983000	-1.61913800	-1.86182300
C	-1.41101000	-2.16699600	-0.74564300
C	1.41116300	-2.16746500	-0.74584400
C	-0.71985500	2.42240800	-0.47137300
C	-1.41087500	2.16693100	0.74559700
C	0.72006500	2.42229300	-0.47134300
C	-1.41037500	1.72897900	-1.50378400
C	-0.71982100	1.61934100	1.86163700
C	-1.41088900	0.43803500	2.24930300
C	0.71996700	1.61925500	1.86157700
C	1.41111800	2.16732900	0.74582500
C	1.41105100	0.43803200	2.24977900
C	0.72003600	-0.80251700	2.33330600
C	-0.71996900	-0.80253400	2.33338300
C	2.60355600	1.32707000	0.45647700
C	2.60347300	0.26790700	1.37764400

C	2.60319300	-1.05925000	0.92122700
C	2.60329500	1.05904600	-0.92105500
C	-0.71986800	0.80274100	-2.33361500
C	0.72006600	0.80270200	-2.33355000
C	1.41064300	1.72933100	-1.50401700
C	-1.41076600	-0.43799700	-2.24921900
C	1.41098800	-0.43804800	-2.24964600
C	2.60352900	-0.26803600	-1.37749500
C	2.60348700	-1.32724500	-0.45638100
C_{60}			
C	1.23569700	-0.01848000	3.31193200
C	2.33652700	0.58614400	2.58788200
C	2.19870400	1.85714000	2.05260300
C	0.95418200	2.58222400	2.21814300
C	-0.09834300	2.00430000	2.91049500
C	0.04594300	0.67473900	3.46981900
C	1.21422100	-1.43278700	2.99512800
C	2.30153700	-1.70217400	2.07499800
C	2.99532800	-0.45437100	1.82343400
C	3.48715800	-0.17804000	0.55742000
C	2.71345600	2.14656800	0.72869000
C	0.69961700	3.31951300	0.99633800
C	-0.59593000	3.44581500	0.52031700
C	-1.69675000	2.84123800	1.24456300
C	-1.45356500	2.13661100	2.41296500
C	-2.14689100	0.88877100	2.66465000
C	-1.22012700	-0.01453900	3.31808700
C	-1.24080500	-1.36693800	3.01532600
C	0.00352300	-2.09178300	2.84981500
C	2.12997900	-2.61925200	1.05000200
C	0.86367700	-3.30848300	0.89781900
C	-0.17575400	-3.05087000	1.77768800
C	-1.53085000	-2.91885300	1.28003200
C	-2.18876800	-1.87770100	2.04479700
C	-3.07472300	-1.01391200	1.42029600
C	-3.05330300	0.40051000	1.73677900
C	-3.30782300	1.13741900	0.51457600
C	-2.64461700	2.33042200	0.27386700
C	1.78677400	3.05007200	0.07589600
C	-1.21422100	1.43278700	-2.99512800
C	-0.00352300	2.09178300	-2.84981500
C	1.24080500	1.36693800	-3.01532600
C	1.22012700	0.01453900	-3.31808700
C	-0.04594300	-0.67473900	-3.46981900
C	-2.33652700	-0.58614400	-2.58788200
C	-2.99532800	0.45437100	-1.82343400
C	-2.30153700	1.70217400	-2.07499800
C	-2.12997900	2.61925200	-1.05000200
C	-0.86367700	3.30848300	-0.89781900
C	0.17575400	3.05087000	-1.77768800
C	2.18876800	1.87770100	-2.04479700
C	3.07472300	1.01391200	-1.42029600
C	3.05330300	-0.40051000	-1.73677900
C	2.14689100	-0.88877100	-2.66465000
C	1.45356500	-2.13661100	-2.41296500
C	0.09834300	-2.00430000	-2.91049500
C	-0.95418200	-2.58222400	-2.21814300
C	-2.19870400	-1.85714000	-2.05260300
C	-3.48715800	0.17804000	-0.55742000
C	-3.34288300	-1.15143200	0.00244400

C	-2.71345600	-2.14656800	-0.72869000
C	-1.78677400	-3.05007200	-0.07589600
C	-0.69961700	-3.31951300	-0.99633800
C	0.59593000	-3.44581500	-0.52031700
C	1.69675000	-2.84123800	-1.24456300
C	2.64461700	-2.33042200	-0.27386700
C	3.30782300	-1.13741900	-0.51457600
C	1.53085000	2.91885300	-1.28003200
C	3.34288300	1.15143200	-0.00244400
C	-1.23569700	0.01848000	-3.31193200
C_{70}			
C	-3.96479100	0.41077500	1.16109500
C	-3.21645200	0.80063500	2.26326400
C	-2.43763000	-0.17024700	2.99455200
C	-2.43746000	-1.48995300	2.60310400
C	-3.21622500	-1.90511700	1.46084800
C	-3.96452200	-0.97732600	0.74943300
C	2.43754800	-2.93625100	-0.61255400
C	3.21624200	-1.97810300	-1.36040400
C	3.96454300	-1.01481100	-0.69785400
C	2.43746900	-1.62250100	-2.52259100
C	2.43762700	-0.32480000	-2.98174700
C	3.21647700	0.68254900	-2.30161900
C	3.96482700	0.35014500	-1.18081200
C	2.43748900	1.89776900	-2.32260400
C	2.43756100	2.73558200	-1.23019400
C	3.21674900	2.40023500	-0.06207300
C	2.43755100	2.79553600	1.08716900
C	2.43747500	2.01532200	2.22142800
C	-1.19944700	-2.24696000	2.61890300
C	0.00000000	-1.69025500	3.11803200
C	0.00000000	-0.28297300	3.53543800
C	-1.19950700	0.45572300	3.42039900
C	1.19958500	1.79658000	2.94624200
C	-1.19958500	1.79658000	2.94624200
C	0.00000000	2.44334900	2.57089100
C	0.00000000	3.27533000	1.36170400
C	-1.19959600	3.39397300	0.62352800
C	-1.19960600	3.35719300	-0.79812000
C	-2.43755500	-2.90066400	0.76347400
C	-2.43754800	-2.93625100	-0.61255400
C	-3.21624200	-1.97810300	-1.36040400
C	-3.96454300	-1.01481100	-0.69785400
C	-2.43746900	-1.62250000	-2.52259000
C	-2.43762700	-0.32480000	-2.98174600
C	-3.21647800	0.68254900	-2.30161900
C	-3.96482800	0.35014500	-1.18081200
C	-2.43748900	1.89776900	-2.32260400
C	-2.43756200	2.73558200	-1.23019400
C	-3.21675000	2.40023500	-0.06207300
C	-3.96482800	1.23135600	-0.03186200
C	-2.43755100	2.79553500	1.08716900
C	-2.43747500	2.01532200	2.22142800
C	3.96479100	0.41077500	1.16109500
C	3.21645200	0.80063500	2.26326400
C	2.43763000	-0.17024700	2.99455200
C	2.43746000	-1.48995300	2.60310400
C	3.21622500	-1.90511700	1.46084800
C	3.96452200	-0.97732700	0.74943300
C	2.43755400	-2.90066400	0.76347300

C	0.00000000	3.20053800	-1.52916200
C	0.00000000	2.30718300	-2.69376200
C	-1.19960000	1.64188300	-3.03515500
C	-1.19951700	0.27833500	-3.43937200
C	0.00000000	-0.46529400	-3.51607000
C	0.00000000	-1.84913300	-3.02651100
C	-1.19945500	-2.37930200	-2.49928400
C	1.19944000	-3.18528400	-1.32758800
C	0.00000000	-3.48820600	-0.64403200
C	0.00000000	-3.45026200	0.82342700
C	1.19944900	-3.11240700	1.49041600
C	1.19944700	-2.24696000	2.61890300
C	1.19950700	0.45572200	3.42039900
C	1.19959600	3.39397300	0.62352900
C	1.19960600	3.35719300	-0.79812000
C	1.19960000	1.64188300	-3.03515500
C	1.19951700	0.27833400	-3.43937200
C	1.19945500	-2.37930200	-2.49928500
C	-1.19944000	-3.18528400	-1.32758800
C	-1.19944900	-3.11240700	1.49041700
C	3.96482800	1.23135600	-0.03186200
C_{84}			
C	-0.38918300	-4.10758400	1.19556800
C	-1.14520900	-4.10052300	0.00000800
C	-0.38918200	-4.10760300	-1.19555100
C	1.02063500	-3.99778100	1.19556200
C	1.76646600	-3.87371800	0.00001700
C	1.02065400	-3.99779600	-1.19552900
C	-2.47159900	-3.46653700	-0.00001300
C	-2.95186400	-2.88279600	-1.19560200
C	-0.87840200	-3.41259400	2.37669300
C	-2.10370400	-2.82689900	2.37662300
C	-2.95187800	-2.88272100	1.19559200
C	2.51619600	-2.46700400	2.37667500
C	1.39634700	-3.23538000	2.37668400
C	0.21560500	-2.76689000	3.04049200
C	3.36275200	-2.39085200	-1.19558300
C	2.97857200	-3.04199700	0.00001900
C	3.36274200	-2.39082800	1.19560200
C	-3.39459100	-0.94556100	2.37669800
C	-3.75195500	-1.71676900	1.19562900
C	-4.12387800	-1.05854200	-0.00001500
C	-3.75195900	-1.71679000	-1.19561000
C	-3.50020500	0.40839700	2.37674300
C	-2.51608700	2.46690800	2.37661000
C	-2.50399100	1.19674700	3.04048100
C	-1.39635800	3.23544600	2.37675300
C	2.50392100	-1.19670700	3.04041300
C	1.31572800	-0.62884400	3.54949200
C	0.11326100	-1.45389600	3.54950500
C	-1.20248400	-0.82506700	3.54948700
C	-2.28836000	-1.57010100	3.04045500
C	1.20247900	0.82510300	3.54949000
C	-0.11330800	1.45396100	3.54959000
C	-1.31572300	0.62891900	3.54949200
C	-0.21561700	2.76693700	3.04055000
C	-3.97249900	1.11497500	1.19552300
C	-4.23804600	0.40707100	-0.00007000
C	-3.97258800	1.11492700	-1.19559100
C	-3.50019400	0.40838300	-2.37671500

C	-3.39459700	-0.94557800	-2.37663900
C	-2.97857200	3.04201300	-0.00009900
C	-3.36281000	2.39087400	-1.19564200
C	-3.36269800	2.39077900	1.19557700
C	-0.87838600	-3.41262400	-2.37666500
C	-2.10369700	-2.82691400	-2.37664400
C	-2.28834700	-1.57011800	-3.04042100
C	-2.50394000	1.19673600	-3.04043600
C	-0.11330400	1.45391600	-3.54953500
C	-1.31570200	0.62890000	-3.54947000
C	-1.20248000	-0.82510300	-3.54945000
C	2.28840500	1.57007500	-3.04047300
C	1.20253100	0.82504700	-3.54948100
C	1.31575600	-0.62885600	-3.54946800
C	0.11326900	-1.45392500	-3.54949200
C	0.21560200	-2.76690600	-3.04046500
C	-1.39632500	3.23535000	-2.37671800
C	-2.51618200	2.46695600	-2.37674700
C	-0.21562100	2.76688300	-3.04049400
C	-1.02063700	3.99777900	1.19556600
C	-1.76643900	3.87374400	-0.00006000
C	-1.02065300	3.99777800	-1.19556800
C	1.39637000	-3.23540000	-2.37664100
C	2.51621600	-2.46704600	-2.37666700
C	2.50397500	-1.19673600	-3.04040100
C	3.75192600	1.71672800	-1.19561700
C	3.39460100	0.94558300	-2.37674700
C	3.50012200	-0.40842100	-2.37666200
C	3.75189400	1.71677200	1.19559400
C	4.12388200	1.05851800	-0.00000900
C	0.87844300	3.41264700	-2.37671700
C	2.95189400	2.88278500	1.19560800
C	2.47157500	3.46651600	-0.00001200
C	2.95190100	2.88277900	-1.19561900
C	2.10371200	2.82692300	-2.37659100
C	2.10365400	2.82690100	2.37660400
C	0.87840800	3.41268700	2.37672100
C	0.38915400	4.10756500	1.19554400
C	1.14521300	4.10057900	-0.00004000
C	0.38924300	4.10754700	-1.19557600
C	3.50013100	-0.40838800	2.37669500
C	3.39456300	0.94558500	2.37669200
C	2.28836200	1.57011000	3.04046100
C	3.97256100	-1.11499100	-1.19554200
C	4.23797200	-0.40707600	0.00000700
C	3.97256100	-1.11496800	1.19557500

Table S8. Coordinates for the $(C_{20})_2$, $(C_{24})_2$, $(C_{36})_2$, $(C_{60})_2$, $(C_{70})_2$ and $(C_{84})_2$ dimers at the equilibrium point.

$(C_{20})_2$			
C	3.47264300	-1.57117800	1.37287100
C	2.53609300	-1.92609400	0.21855600
C	2.99096900	-0.38017300	1.94434100
C	1.81628000	0.02109500	1.21269700
C	1.54711200	-0.92617300	0.16430000
C	4.00498300	0.70595000	1.82510700
C	3.39601600	1.72177700	0.95946600
C	2.06284300	1.30540300	0.61847200
C	5.08672900	0.18368500	1.12042300
C	5.13196600	0.90498100	-0.18514100
C	4.14096900	1.90271300	-0.23919500
C	4.71598700	-1.19731700	0.78725800
C	4.61633100	-1.32860600	-0.63988500
C	4.85923500	-0.04288400	-1.23303900
C	3.28079600	-1.74313700	-0.97905600
C	2.67254100	-0.72796000	-1.84633300
C	3.68626200	0.35755000	-1.96697500
C	3.20410000	1.54796300	-1.39363400
C	1.96046600	1.17653800	-0.80932200
C	1.59193100	-0.20441200	-1.14158300
C	-1.76529400	-1.36870900	-0.13724800
C	-3.13136500	-2.04350100	-0.27951500
C	-1.79983500	-0.65418500	1.08787800
C	-3.07382300	-0.87365700	1.71982600
C	-3.88455000	-1.70341400	0.87134400
C	-1.60783800	0.79262400	0.88661500
C	-2.86772700	1.42300300	1.36054900
C	-3.72801200	0.39496400	1.87973300
C	-1.56926700	0.98431900	-0.49266400
C	-2.79211900	1.72632700	-0.85111300
C	-3.54432900	2.06342700	0.30111500
C	-1.71717100	-0.35149900	-1.11545700
C	-2.94951900	-0.37309600	-1.85956900
C	-3.60448800	0.89556100	-1.69830400
C	-3.80816400	-1.40076300	-1.33788600
C	-5.06843300	-0.77056100	-0.86462100
C	-4.87868600	0.67724100	-1.06813800
C	-4.91118900	1.39040000	0.15851300
C	-4.96209000	0.37447700	1.13654100
C	-5.11035200	-0.96268200	0.51307400
$(C_{24})_2$			
C	-1.81638200	-1.23963200	1.01759000
C	-1.86447800	0.05971400	1.68330400
C	-2.96285100	-1.91056100	1.28239600
C	-3.28150300	0.24789200	2.21548200
C	-3.95350100	-0.97573000	1.97297600
C	-3.56528700	-2.32598400	0.01825300
C	-4.85740800	-1.91907600	-0.00557400
C	-5.12561600	-0.99442600	1.17971600
C	-2.79123500	-1.64022900	-1.10455700
C	-1.70474300	-0.98034100	-0.48045500
C	-1.55574700	0.42106200	-0.62191900
C	-3.72520700	-0.90041500	-1.87074000
C	-3.57274600	0.50061400	-2.01126700
C	-2.48589700	1.16252900	-1.38910700
C	-5.08542900	-1.14525500	-1.22245800
C	-2.65592300	2.01899100	0.80149500

C	-1.58274400	1.02811600	0.77791800
C	-3.10463400	2.20575100	-0.46280600
C	-4.55182900	2.00695000	-0.48593300
C	-4.85597700	1.12096900	-1.46425200
C	-5.65130500	0.03939000	-0.88844100
C	-3.78142500	1.45154400	1.66322400
C	-4.95512900	1.43389300	0.86981500
C	-5.62593900	0.21098900	0.62737800
C	5.24176200	-0.95370200	-1.34628700
C	5.79003000	0.20219300	-0.64043800
C	4.85614500	-1.87989100	-0.43613000
C	5.49452000	0.01234500	0.84306600
C	4.92829600	-1.27960700	0.96605000
C	3.46254500	-2.23550900	-0.68785200
C	2.76192900	-2.11326800	0.46544300
C	3.63346700	-1.43340500	1.51962000
C	2.92199200	-1.25547600	-1.72603200
C	4.02970500	-0.47193300	-2.13740900
C	3.99862300	0.93492800	-1.98555700
C	1.78637100	-0.63054200	-1.16071600
C	1.75286100	0.77816400	-1.00990100
C	2.85931800	1.55964800	-1.41994800
C	1.60994900	-1.24744600	0.22568700
C	4.63300700	2.05221000	0.05097400
C	5.17419000	1.32177100	-1.09072600
C	3.32361200	2.31621800	-0.17749700
C	2.53944000	1.82624000	0.95251600
C	1.54903100	1.03287700	0.47900100
C	1.61278200	-0.26158200	1.15488000
C	4.76895400	1.15060600	1.27594300
C	3.47592200	0.99552400	1.82945600
C	2.90848200	-0.29711700	1.95381600
$(C_{36})_2$			
C	5.00069600	1.21294300	-2.06976200
C	5.33105400	-1.57848300	-1.82479000
C	6.18108900	-0.71365300	-1.07849900
C	6.01271900	0.71027900	-1.20463100
C	4.36831900	2.40201900	-1.43769400
C	2.97749100	2.23019200	-1.52080600
C	2.19801400	2.24178400	-0.35330500
C	4.97846100	2.58385400	-0.18740100
C	4.20027500	2.59640300	0.97984200
C	2.80997100	2.42452300	0.89678700
C	6.01125600	-0.51643500	1.37544300
C	5.84303800	0.90747800	1.24889400
C	5.99741400	1.51017400	-0.02972700
C	6.32938700	-1.27939800	0.21691700
C	1.61400900	0.38524100	0.99539700
C	1.46113500	0.95222500	-0.30089200
C	1.78065900	-1.03821800	1.12218600
C	2.45873200	1.25029500	1.73940500
C	1.77941700	0.18862800	-1.45695300
C	2.73148300	0.93373000	-2.20610100
C	1.94732300	-1.23580600	-1.33010600
C	1.78579800	-1.84107800	-0.05247400
C	3.05897600	-1.85934600	-1.96014900
C	4.14900400	-1.07357300	-2.43110800
C	3.98111800	0.35106800	-2.55818700
C	2.80649000	-2.91027300	0.10413100
C	3.58517300	-2.92185100	-1.06383100

C	4.97626200	-2.75019000	-0.98097700
C	3.41876900	-2.72819300	1.35451600
C	3.64134800	0.74572600	2.35078000
C	3.80926500	-0.67857000	2.47710900
C	2.78750900	-1.54101300	1.98952900
C	4.72668200	1.52958500	1.87442800
C	5.05773700	-1.26068000	2.12362700
C	4.80936600	-2.55664800	1.43670200
C	5.58806100	-2.56775100	0.26888600
C	-3.05206900	1.84178600	-1.88962800
C	-2.74146200	-0.95475600	-2.11772400
C	-1.78381300	-0.21021200	-1.37482300
C	-1.94324200	1.21596600	-1.25715900
C	-3.57075300	2.91322700	-0.99951800
C	-4.96270400	2.75026300	-0.91352800
C	-5.57368800	2.57944700	0.33837200
C	-2.79040400	2.90477000	0.16736500
C	-3.40181000	2.73448000	1.41979900
C	-4.79324500	2.57153000	1.50505500
C	-1.61519800	-0.39160400	1.07866700
C	-1.77314400	1.03365600	1.19629900
C	-1.77623900	1.82857200	0.01633200
C	-1.46846300	-0.96789000	-0.21439600
C	-6.00681700	0.53805800	1.45925500
C	-6.32256900	1.29520600	0.29601300
C	-5.84706400	-0.88764800	1.34194000
C	-5.04811400	1.28166700	2.20094500
C	-6.17932100	0.72014300	-0.99568600
C	-5.32556600	1.57517500	-1.74924400
C	-6.01943500	-0.70561400	-1.11262600
C	-6.00707900	-1.49788300	0.06766600
C	-5.01204300	-1.21992500	-1.97603000
C	-3.98799200	-0.36714500	-2.47163900
C	-4.14741700	1.05927500	-2.35372700
C	-4.99473200	-2.57843500	-0.08454100
C	-4.38560400	-2.40838500	-1.33705300
C	-2.99398200	-2.24520100	-1.42349900
C	-4.21465800	-2.58778900	1.08150000
C	-3.80218200	0.69469100	2.55640300
C	-3.64272500	-0.73133500	2.43929400
C	-4.73331800	-1.51204600	1.96991500
C	-2.77641600	1.54785300	2.06165700
C	-2.46402900	-1.24675800	1.82937100
C	-2.82351600	-2.42458400	0.99498200
C	-2.21268500	-2.25354000	-0.25719800
$(C_{60})_2$			
C	6.85540300	3.15974500	-0.02038100
C	5.70588900	3.54806800	-0.81377400
C	4.46213100	3.67615400	-0.21574500
C	4.31036700	3.42086000	1.20318800
C	5.40941000	3.04954500	1.96181700
C	6.71042800	2.91597400	1.33625300
C	7.68412300	2.28801300	-0.82934800
C	7.04715500	2.13701300	-2.12269300
C	5.82469700	2.91631300	-2.11300900
C	4.69401900	2.43975700	-2.75733400
C	3.27962100	3.17783500	-0.89000500
C	3.03399300	2.76470400	1.40632100
C	2.91247300	1.76600700	2.35969200
C	4.06164200	1.37744100	3.15313700

C	5.28215800	2.00484200	2.95864500
C	6.50436600	1.22543000	2.94920100
C	7.38705500	1.78855300	1.94651900
C	8.17917600	0.95506600	1.17295600
C	8.33138600	1.21031500	-0.24568300
C	7.08527000	0.91544400	-2.77607400
C	7.76223900	-0.21160000	-2.16569600
C	8.37124800	-0.06750300	-0.92898600
C	8.24381800	-1.11243200	0.06761700
C	8.12508400	-0.48049400	1.36659800
C	7.28069800	-1.01904900	2.32463800
C	6.45222000	-0.14725900	3.13386400
C	5.17600800	-0.80358600	3.33683800
C	4.00738400	-0.05849400	3.34679700
C	2.39700900	2.61460200	0.11226700
C	2.22734900	-1.93041500	0.72608800
C	1.58627400	-0.85084900	0.14169100
C	1.73411400	-0.59606600	-1.27691800
C	2.52329000	-1.43047700	-2.05109200
C	3.19979500	-2.55824000	-1.44023400
C	4.20426400	-3.19062600	0.70990400
C	4.08530100	-2.55859900	2.00942300
C	2.86287900	-1.77941800	2.01960600
C	2.82473400	-0.55747500	2.67319600
C	2.14919400	0.56980200	2.06275800
C	1.54634100	0.42565700	0.82429100
C	1.78583000	0.83910300	-1.47105800
C	2.62904400	1.37789000	-2.42962000
C	3.45754800	0.50575200	-3.23883200
C	3.40570300	-0.86715900	-3.05385900
C	4.62799100	-1.64634800	-3.06255800
C	4.50062300	-2.69134700	-2.06560100
C	5.59967800	-3.06262900	-1.30696300
C	5.44807000	-3.31797900	0.11165400
C	5.21587100	-2.08138400	2.65329500
C	6.51667400	-2.21464900	2.02771100
C	6.63049300	-2.81892000	0.78567000
C	7.51339400	-2.25561200	-0.21637100
C	6.87623000	-2.40604900	-1.50980100
C	6.99794000	-1.40706600	-2.46263400
C	5.84843700	-1.01873700	-3.25632500
C	5.90257800	0.41680200	-3.45006200
C	4.73387000	1.16189500	-3.44092000
C	1.66973800	1.47052800	-0.17206100
C	3.39310100	2.57366600	-2.13240400
C	3.05509500	-2.80259600	-0.08352500
C	-3.05449200	2.80221400	0.08386800
C	-4.20362800	3.19058200	-0.70944700
C	-5.44738400	3.31809200	-0.11112000
C	-5.59897300	3.06256600	1.30746700
C	-4.49995500	2.69095900	2.06600100
C	-3.19917100	2.55768700	1.44054900
C	-2.22696200	1.92997800	-0.72591200
C	-2.86258900	1.77929800	-2.01941500
C	-4.08485400	2.55872800	-2.00906400
C	-5.21555400	2.08183300	-2.65294100
C	-6.62994100	2.81936800	-0.78514400
C	-6.87564400	2.40620100	1.51027000
C	-6.99750800	1.40711000	2.46296700
C	-5.84803500	1.01843300	3.25654800

C	-4.62747900	1.64583500	3.06281000
C	-3.40534300	0.86640900	3.05393600
C	-2.52286100	1.42970200	2.05121800
C	-1.73389500	0.59524400	1.27688700
C	-1.58609200	0.85020000	-0.14169500
C	-2.82472000	0.55744600	-2.67318800
C	-2.14937500	-0.57004800	-2.06295300
C	-1.54641500	-0.42620600	-0.82450300
C	-1.66995100	-1.47119300	0.17172000
C	-1.78586900	-0.83994100	1.47082900
C	-2.62916400	-1.37869800	2.42934100
C	-3.45745600	-0.50651900	3.23872000
C	-4.73389800	-1.16243600	3.44076300
C	-5.90245700	-0.41711800	3.45007300
C	-7.51290100	2.25607900	0.21685000
C	-7.68451400	-2.28759000	0.82916500
C	-8.33159300	-1.20967900	0.24568600
C	-8.17939900	-0.95425000	-1.17292600
C	-7.38747600	-1.78778100	-1.94664400
C	-6.71105000	-2.91542500	-1.33657300
C	-5.70652200	-3.54802500	0.81331800
C	-5.82514200	-2.91643800	2.11264900
C	-7.04744800	-2.13689900	2.12249900
C	-7.08528400	-0.91542100	2.77606200
C	-7.76205800	0.21184100	2.16588700
C	-8.37116100	0.06804200	0.92918600
C	-8.12502800	0.48132500	-1.36635700
C	-7.28058100	1.01986300	-2.32435500
C	-6.45231600	0.14803000	-3.13375000
C	-6.50473000	-1.22467800	-2.94927800
C	-5.28267500	-2.00432800	-2.95890000
C	-5.41007800	-3.04915700	-1.96221700
C	-4.31107500	-3.42079200	-1.20369000
C	-4.46281600	-3.67625900	0.21521400
C	-4.69433600	-2.44020000	2.75698300
C	-3.39347400	-2.57427100	2.13197700
C	-3.28017600	-3.17827000	0.88948400
C	-2.39750800	-2.61505900	-0.11273600
C	-3.03458500	-2.76485000	-1.40678300
C	-2.91291000	-1.76605400	-2.36003100
C	-4.06205000	-1.37713800	-3.15336400
C	-4.00750900	0.05880800	-3.34680900
C	-5.17598300	0.80413100	-3.33668100
C	-8.24357000	1.11309000	-0.06727300
C	-6.51630300	2.21526500	-2.02728100
C	-6.85600200	-3.15936800	0.02003300
$(C_{70})_2$			
C	-4.67977100	3.96617700	1.17076400
C	-4.31714300	3.21790400	2.28242400
C	-5.30572900	2.43895500	2.98978800
C	-6.61538300	2.43855700	2.56615900
C	-7.00245400	3.21733000	1.41405100
C	-6.05758500	3.96572500	0.72531700
C	-7.98314300	-2.43694300	-0.68334300
C	-7.00704800	-3.21567000	-1.40774000
C	-6.05996700	-3.96405000	-0.72206200
C	-6.62367200	-2.43689500	-2.56109800
C	-5.31539300	-2.43727200	-2.98897500
C	-4.32450900	-3.21620900	-2.28482000
C	-4.68359400	-3.96451800	-1.17205600

C	-3.10928400	-2.43707600	-2.27706400
C	-2.29896500	-2.43688500	-1.16392300
C	-2.66166500	-3.21635300	-0.00373500
C	-2.29526000	-2.43675900	1.15519900
C	-3.10197700	-2.43684200	2.27096700
C	-7.37292100	1.20061000	2.56384100
C	-6.82880900	0.00098700	3.07663700
C	-5.43187800	0.00101100	3.52824800
C	-4.69066900	1.20083200	3.43118000
C	-3.33856600	-1.19894400	2.99038800
C	-3.33856700	1.20091000	2.99026300
C	-2.68355400	0.00096400	2.63047100
C	-1.82661000	0.00090300	1.44018900
C	-1.69128500	1.19967300	0.70516400
C	-1.69348700	1.19961200	-0.71624600
C	-7.98089800	2.43861100	0.69281500
C	-7.98314300	2.43853000	-0.68359400
C	-7.00704900	3.21718300	-1.40807100
C	-6.05996800	3.96563400	-0.72247000
C	-6.62367200	2.43829000	-2.56134800
C	-5.31539300	2.43862400	-2.98922600
C	-4.32450900	3.21763400	-2.28515200
C	-4.68359500	3.96605800	-1.17246500
C	-3.10928400	2.43850200	-2.27731600
C	-2.29896500	2.43842400	-1.16417500
C	-2.66166500	3.21801200	-0.00406700
C	-3.83037100	3.96675100	-0.00222600
C	-2.29526000	2.43853600	1.15494800
C	-3.10197600	2.43873400	2.27071600
C	-4.67977100	-3.96439700	1.17117200
C	-4.31714300	-3.21601100	2.28275600
C	-5.30572900	-2.43698900	2.99004000
C	-6.61538300	-2.43663400	2.56641100
C	-7.00245400	-3.21552600	1.41438200
C	-6.05758500	-3.96399200	0.72572500
C	-7.98089800	-2.43688100	0.69306600
C	-1.83110600	0.00075400	-1.45072900
C	-2.69199600	0.00069400	-2.63821500
C	-3.34821800	1.20059400	-2.99596500
C	-4.70177300	1.20046900	-3.43246700
C	-5.44329600	0.00064700	-3.52698800
C	-6.83874900	0.00067100	-3.07087600
C	-7.38120000	1.20034700	-2.55644600
C	-8.21511100	-1.19878700	-1.40426300
C	-8.53445400	0.00079100	-0.72842600
C	-8.53207800	0.00086700	0.73966900
C	-8.21054000	-1.19864400	1.41459900
C	-7.37292100	-1.19868800	2.56396500
C	-4.69067000	-1.19882000	3.43130400
C	-1.69128400	-1.19794300	0.70529000
C	-1.69348500	-1.19802900	-0.71612300
C	-3.34821700	-1.19924300	-2.99584100
C	-4.70177300	-1.19916500	-3.43234400
C	-7.38120100	-1.19895300	-2.55632400
C	-8.21511000	1.20030000	-1.40438700
C	-8.21054200	1.20044800	1.41447600
C	-3.83037100	-3.96509300	-0.00181800
C	4.67977100	-3.96617700	1.17076400
C	4.31714300	-3.21790400	2.28242400
C	5.30572900	-2.43895500	2.98978800

C	6.61538300	-2.43855700	2.56615900
C	7.00245400	-3.21733000	1.41405100
C	6.05758500	-3.96572500	0.72531700
C	7.98314300	2.43694300	-0.68334300
C	7.00704800	3.21567000	-1.40774000
C	6.05996700	3.96405000	-0.72206200
C	6.62367200	2.43689500	-2.56109800
C	5.31539300	2.43727200	-2.98897500
C	4.32450900	3.21620900	-2.28482000
C	4.68359400	3.96451800	-1.17205600
C	3.10928400	2.43707600	-2.27706400
C	2.29896500	2.43688500	-1.16392300
C	2.66166500	3.21635300	-0.00373500
C	2.29526000	2.43675900	1.15519900
C	3.10197700	2.43684200	2.27096700
C	7.37292100	-1.20061000	2.56384100
C	6.82880900	-0.00098700	3.07663700
C	5.43187800	-0.00101100	3.52824800
C	4.69066900	-1.20083200	3.43118000
C	3.33856600	1.19894400	2.99038800
C	3.33856700	-1.20091000	2.99026300
C	2.68355400	-0.00096400	2.63047100
C	1.82661000	-0.00090300	1.44018900
C	1.69128500	-1.19967300	0.70516400
C	1.69348700	-1.19961200	-0.71624600
C	7.98089800	-2.43861100	0.69281500
C	7.98314300	-2.43853000	-0.68359400
C	7.00704900	-3.21718300	-1.40807100
C	6.05996800	-3.96563400	-0.72247000
C	6.62367200	-2.43829000	-2.56134800
C	5.31539300	-2.43862400	-2.98922600
C	4.32450900	-3.21763400	-2.28515200
C	4.68359500	-3.96605800	-1.17246500
C	3.10928400	-2.43850200	-2.27731600
C	2.29896500	-2.43842400	-1.16417500
C	2.66166500	-3.21801200	-0.00406700
C	3.83037100	-3.96675100	-0.00222600
C	2.29526000	-2.43853600	1.15494800
C	3.10197600	-2.43873400	2.27071600
C	4.67977100	3.96439700	1.17117200
C	4.31714300	3.21601100	2.28275600
C	5.30572900	2.43698900	2.99004000
C	6.61538300	2.43663400	2.56641100
C	7.00245400	3.21552600	1.41438200
C	6.05758500	3.96399200	0.72572500
C	7.98089800	2.43688100	0.69306600
C	1.83110600	-0.00075400	-1.45072900
C	2.69199600	-0.00069400	-2.63821500
C	3.34821800	-1.20059400	-2.99596500
C	4.70177300	-1.20046900	-3.43246700
C	5.44329600	-0.00064700	-3.52698800
C	6.83874900	-0.00067100	-3.07087600
C	7.38120000	-1.20034700	-2.55644600
C	8.21511100	1.19878700	-1.40426300
C	8.53445400	-0.00079100	-0.72842600
C	8.53207800	-0.00086700	0.73966900
C	8.21054000	1.19864400	1.41459900
C	7.37292100	1.19868800	2.56396500
C	4.69067000	1.19882000	3.43130400
C	1.69128400	1.19794300	0.70529000

C	1.69348500	1.19802900	-0.71612300
C	3.34821700	1.19924300	-2.99584100
C	4.70177300	1.19916500	-3.43234400
C	7.38120100	1.19895300	-2.55632400
C	8.21511000	-1.20030000	-1.40438700
C	8.21054200	-1.20044800	1.41447600
C	3.83037100	3.96509300	-0.00181800
$(C_{84})_2$			
C	1.22457700	-4.10736300	0.38933300
C	0.02892900	-4.10096200	1.14518400
C	-1.16665500	-4.10890400	0.38905800
C	1.22453200	-3.99751000	-1.02089800
C	0.02884900	-3.87417700	-1.76670800
C	-1.16668700	-3.99908500	-1.02073400
C	0.02822700	-3.46701700	2.47171000
C	-1.16795700	-2.88401300	2.95184700
C	2.40465900	-3.41133100	0.87901300
C	2.40406500	-2.82567900	2.10450200
C	1.22316700	-2.88228100	2.95254100
C	2.40391300	-2.46565300	-2.51684500
C	2.40469100	-3.23408200	-1.39687400
C	3.06617400	-2.76387300	-0.21541500
C	-1.16811900	-2.39210000	-3.36290800
C	0.02807900	-3.04236500	-2.97888900
C	1.22299300	-2.39027300	-3.36345700
C	2.40206900	-0.94336400	3.39596000
C	1.22193100	-1.71595100	3.75283500
C	0.02573300	-1.05859500	4.12423500
C	-1.16913800	-1.71805000	3.75189200
C	2.40071000	0.41078400	3.50144700
C	2.39868000	2.47041200	2.51707200
C	3.06200400	1.19982300	2.50415400
C	2.39804100	3.23884700	1.39710900
C	3.06471000	-1.19373600	-2.50387200
C	3.56556200	-0.62469400	-1.31432400
C	3.56642800	-1.44904600	-0.11299800
C	3.56565500	-0.82074000	1.20153000
C	3.06492900	-1.56710200	2.28868500
C	3.56414000	0.82784300	-1.20118000
C	3.56351200	1.45615500	0.11335000
C	3.56418100	0.63179700	1.31467800
C	3.06062400	2.76999300	0.21572000
C	1.21912100	1.11641100	3.97351100
C	0.02424800	0.40710500	4.23838100
C	-1.17204700	1.11385100	3.97253000
C	-2.35228300	0.40604900	3.49991100
C	-2.35090600	-0.94795500	3.39443600
C	0.02167300	3.04236600	2.97888900
C	-1.17326500	2.38970100	3.36278300
C	1.21784000	2.39266600	3.36357600
C	-2.34829100	-3.41489200	0.87827700
C	-2.34898900	-2.82930300	2.10363300
C	-3.01408300	-1.57311400	2.28825900
C	-3.01678600	1.19366800	2.50374000
C	-3.52605400	1.45037100	0.11309400
C	-3.52526600	0.62526700	1.31555200
C	-3.52382200	-0.82861800	1.20229700
C	-3.01700100	1.56701300	-2.28857500
C	-3.52535600	0.82149600	-1.20266200
C	-3.52386900	-0.63239300	-1.31591600

C	-3.52312300	-1.45749500	-0.11346000
C	-3.01272900	-2.76985700	-0.21571500
C	-2.35508200	3.23296400	1.39619500
C	-2.35429500	2.46461700	2.51604000
C	-3.01830500	2.76375500	0.21540000
C	1.21640000	3.99991200	1.02101400
C	0.02089600	3.87417300	1.76670500
C	-1.17481300	3.99667900	1.02060700
C	-2.34844300	-3.23773200	-1.39644200
C	-2.34908400	-2.46938400	-2.51628600
C	-3.01412200	-1.19977300	-2.50405000
C	-1.17220300	1.71564800	-3.75201900
C	-2.35245800	0.94318300	-3.39468300
C	-2.35110200	-0.41081800	-3.50015500
C	1.21886500	1.71834200	-3.75272400
C	0.02402500	1.05858900	-4.12424300
C	-2.35505000	3.41012000	-0.87852400
C	1.21767700	2.88467300	-2.95242900
C	0.02152400	3.46701500	-2.47172100
C	-1.17344000	2.88161200	-2.95197600
C	-2.35445000	2.82453000	-2.10388100
C	2.39858600	2.83043800	-2.10426100
C	2.39789000	3.41609600	-0.87877500
C	1.21636700	4.10976300	-0.38921900
C	0.02081500	4.10095700	-1.14519300
C	-1.17485800	4.10649600	-0.38918500
C	2.40189600	-0.40602700	-3.50121200
C	2.40051500	0.94812500	-3.39573300
C	3.06198100	1.57318700	-2.28837200
C	-1.16939500	-1.11625000	-3.97265700
C	0.02550900	-0.40711000	-4.23838500
C	1.22177700	-1.11402100	-3.97339400
C	11.66667900	-4.10889100	0.38907200
C	10.47109400	-4.10095600	1.14519600
C	9.27544800	-4.10736500	0.38934300
C	11.66671300	-3.99907400	-1.02072000
C	10.47117800	-3.87417400	-1.76669600
C	9.27549400	-3.99751400	-1.02088800
C	10.47179100	-3.46700900	2.47172100
C	9.27684600	-2.88227900	2.95255000
C	12.84831000	-3.41487200	0.87829200
C	12.84900300	-2.82928000	2.10364800
C	11.66796900	-2.88399800	2.95185900
C	12.84910400	-2.46936900	-2.51627200
C	12.84846500	-3.23771500	-1.39642800
C	13.51274600	-2.76983400	-0.21569900
C	9.27702800	-2.39028000	-3.36344900
C	10.47194500	-3.04236500	-2.97887900
C	11.66814000	-2.39209300	-3.36289700
C	12.85090500	-0.94793100	3.39444800
C	11.66914200	-1.71803200	3.75190200
C	10.47426600	-1.05858400	4.12424300
C	9.27807200	-1.71594900	3.75284100
C	12.85227300	0.40607300	3.49992100
C	12.85427600	2.46464100	2.51604800
C	13.51677400	1.19369600	2.50375100
C	12.85505800	3.23298400	1.39620100
C	13.51413400	-1.19975300	-2.50403700
C	14.02387500	-0.63236700	-1.31590200
C	14.02313100	-1.45746800	-0.11344500

C	14.02382500	-0.82859000	1.20231200
C	13.51408900	-1.57308800	2.28827400
C	14.02535500	0.82152100	-1.20265100
C	14.02604600	1.45039800	0.11310600
C	14.02526200	0.62529600	1.31556400
C	13.51828800	2.76378000	0.21540900
C	11.67203300	1.11386700	3.97253700
C	10.47574200	0.40711500	4.23838700
C	9.28086500	1.11641500	3.97351400
C	8.09928100	0.41077900	3.50144800
C	8.09793000	-0.94336900	3.39596300
C	10.47830400	3.04237500	2.97889100
C	9.28213900	2.39266700	3.36357700
C	11.67324400	2.38971800	3.36278800
C	8.09536100	-3.41134000	0.87901900
C	8.09594900	-2.82568600	2.10450800
C	7.43507700	-1.56711300	2.28868800
C	7.43798400	1.19981100	2.50415200
C	6.93647900	1.45613800	0.11334700
C	6.93581300	0.63178100	1.31467600
C	6.93434800	-0.82075600	1.20153000
C	7.43801400	1.57317000	-2.28837300
C	6.93585800	0.82782500	-1.20118100
C	6.93444400	-0.62471400	-1.31432300
C	6.93358100	-1.44906300	-0.11299700
C	7.43384300	-2.76388800	-0.21541100
C	8.10193500	3.23883900	1.39710700
C	8.10130200	2.47040500	2.51706900
C	7.43935800	2.76998000	0.21571600
C	11.67478500	3.99669200	1.02061000
C	10.47907700	3.87418000	1.76670600
C	9.28357200	3.99991000	1.02101200
C	8.09533100	-3.23409400	-1.39686700
C	8.09610700	-2.46566600	-2.51684000
C	7.43530200	-1.19375400	-2.50386900
C	9.28113200	1.71833500	-3.75272200
C	8.09948600	0.94811000	-3.39573300
C	8.09811300	-0.40604100	-3.50121100
C	11.67219900	1.71565500	-3.75201300
C	10.47597700	1.05858800	-4.12423800
C	8.10209000	3.41608400	-0.87877800
C	11.67342700	2.88162100	-2.95197200
C	10.47845900	3.46701700	-2.47171900
C	9.28231100	2.88466600	-2.95242900
C	8.10140000	2.83042500	-2.10426300
C	12.85443600	2.82454800	-2.10387400
C	12.85503000	3.41013800	-0.87851800
C	11.67483300	4.10650800	-0.38918300
C	10.47916200	4.10096000	-1.14519200
C	9.28360800	4.10976000	-0.38921900
C	12.85111100	-0.41080400	-3.50014300
C	12.85246000	0.94319800	-3.39467400
C	13.51699600	1.56703400	-2.28856600
C	9.27823800	-1.11402900	-3.97338800
C	10.47450100	-0.40711100	-4.23837800
C	11.66941000	-1.11624400	-3.97264700

References

[1] L.A. Girifalco, Molecular properties of fullerene in the gas and solid phases, J. Phys. Chem. 96 (1992) 858–861. https://doi.org/10.1021/j100181a061.

[2] F.-L. Liu, J. Wang, The intermolecular potential function of Smith–Thakkar type for C60, J. Mol. Struct. THEOCHEM. 778 (2006) 105–109. https://doi.org/https://doi.org/10.1016/j.theochem.2006.06.012.

[3] T.-C. Lim, Preliminary assessment of a multifunctional potential energy function, Mol. Phys. 108 (2010) 1589–1597. https://doi.org/10.1080/00268976.2010.484817.
